# Long-term ocular symptoms following COVID-19 linked to immune dysregulation, dysautonomia and peripheral neuropathy

**DOI:** 10.1038/s41467-026-74858-4

**Published:** 2026-07-08

**Authors:** Petros Moustardas, Helen Setterud, Helena Meijer, Gunnel Andersson, Jenny Roth, Ava Dashti, Björn Johansson, António Filipe Macedo, Neil Lagali

**Affiliations:** 1 Division of Ophthalmology, Department of Biomedical and Clinical Sciences, Faculty of Medicine, Linköping University, Linköping, Sweden; 2 Clinical Department of Ophthalmology, Region Östergötland, Linköping, Sweden; 3 Department of Medicine and Optometry, Linnaeus University, Kalmar, Sweden; 4 Department and Centre of Physics-Optometry and Vision Science, University of Minho, Braga, Portugal; 5 Department of Ophthalmology, Sørlandet Hospital Arendal, Arendal, Norway

**Keywords:** Viral infection, Peripheral neuropathies, Autonomic nervous system, Diagnostic markers

## Abstract

COVID-19 does not require hospitalization in most cases, but post-acute sequelae can persist and are a public health concern. In a prospective cross-sectional study, we examine persistent ocular symptoms (POS) emerging in non-hospitalized individuals after COVID-19, with individuals without POS post-recovery as controls. Using symptom and quality of life data, clinical examinations and biofluid proteomics, we document ocular symptoms persisting from 3 months up to 3 years post-infection. POS lead to significant vision disability and are linked to clinical findings not detectable in routine exams but only with specialized tests. POS include near vision disturbances, strabismus, weakened autonomic pupillary reflexes, corneal neurodegeneration and chronic activation of ocular surface dendritic/T cells. We report a tear film proteomic profile consistent with severe COVID-19, chronic dysregulation of CD4^+^ T cell regulatory activity, upregulation of ITGB6, NFASC, CTGF, TPSAB1 and CKMT1A-CKMT1B, pupil dysfunction correlating with elevated JUN, and dendritic/T cell dysregulation correlating with elevated ANGPTL2, SKAP2 and DAPP1 levels. Diagnostic models based on clinical examinations with or without biomarkers predict POS with 77-91% accuracy and implicate chronic T cell-mediated neuroinflammation in the pathogenesis of POS, a debilitating syndrome arising after COVID-19 recovery and characterized by strabismus, ocular dysautonomia and peripheral ocular neuropathy.

## Abstract (web summary)

The authors studied Swedish individuals experiencing persistent ocular and vision symptoms after COVID-19. Special exams found ocular markers consistent with severe COVID-19. They documented broad symptomatic burden extending for years after infection.

## Introduction

During and following SARS-CoV-2 infection, loss of smell and taste are the most widely reported sensory symptoms^1,2^. Although less widely known, ocular symptoms have up to 31–35% estimated prevalence up to 7 months post-infection in severe COVID-19 and long-COVID^3,4^, with ocular pain, light sensitivity, reduced/blurred vision, and difficulties in reading most frequently reported, and with women overrepresented in long-COVID cohorts^3,5^. In addition, effects in the posterior eye have been widely reported after COVID-19^6,7^. Ocular pathology from SARS-CoV-2 infection, however, lacks diagnostic criteria and biomarkers, hindering awareness, diagnosis, and medical management. Furthermore, whether ocular sequelae persist for months after COVID-19 recovery is unknown. Given the large global population having had COVID-19^8^ with incompletely documented post-acute sequelae, a potentially large population may experience ocular symptoms.

The aim of this study was to identify, formally characterize, and report common pathologic modalities between people who experience persistent (> 12 weeks) ocular symptoms (POS) after recovery from COVID-19 using an exhaustive battery of clinical tests, and to screen for biomarkers/mechanistic insights using tear film proteomics and enrichment analysis. In addition, we aimed to develop diagnostic models that can accurately identify and classify POS, potentially able to be deployed after normalization according to the specific needs and conditions in eye clinics.

Here, we report a distinct array of ocular symptoms that define post-COVID POS leading to significant vision disability, quantified via the Catquest-9SF survey and linked to clinical findings not detectable in routine exams but only with specialized tests. Furthermore, we identify key protein marker dysregulation in the tear film of POS subjects, highlight T cell-mediated neuroinflammation, dysautonomia, and peripheral ocular neuropathy as characteristic traits of POS, and develop two diagnostic models to objectively discriminate POS symptomatology.

## Results

### Study cohort

**Fig. 1:**
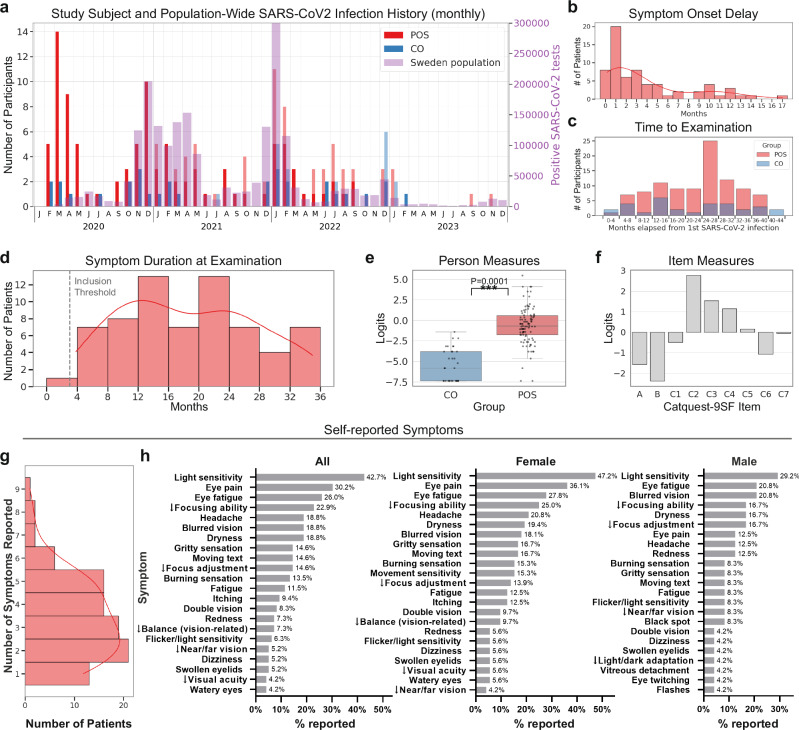
Clinical course characteristics of study participants and long-term debilitating ocular symptoms. **a** Distribution of SARS-CoV-2 infection times in study participants. Instances of subsequent infections are counted on top of the first infection instances, and shaded with a lighter color. Purple bars represent country-wide Positive SARS-CoV-2 cases reported in the Swedish Health Authority database. **b** Delay of persistent ocular symptom (POS) onset from first SARS-CoV-2 infection (red line, kernel density estimation). **c** Time elapsed from first infection to study examination, and **d** POS duration at examination. **e** Aggregate Catquest-9SF person measure difficulty score for vision in daily life (positive values indicate increased disability). Boxes represent the interquartile range (IQR, 25th–75th percentile), center lines represent the median, whiskers extend to the most extreme values within 1.5×IQR, and dots represent individual participants. Detailed numeric values for the box plots and group sizes are provided as a .csv file in the Source Data container file. Two-tailed group-wise *t* test *P* value is reported. **f** Catquest-9SF responses per question indicating level of difficulty experienced by all respondents (negative values indicate increased difficulty). **g** Distribution of number of POS reported from 96 free-text responses and **h** prevalence of distinct self-reported symptoms in the full POS group and in females (*n* = 72) and males (*n* = 24). Source data for panels abcdeg are provided as separate .csv files in the Source Data container file. POS persistent ocular symptoms group, CO control group.

In this study, we prospectively examined 100 individuals in Sweden who developed POS post-COVID-19 that did not require hospitalization, and 32 control (CO) subjects who recovered from infection without developing POS or requiring hospitalization (Supplementary Table 1). Of the recruited participants with POS, 73% were female, and six participants were under the age of 18 at the time of examination. Infections closely matched the timeline of documented national cases after testing became widely available (Fig. 1a). Onset of POS was 0.2–4 months after initial infection in 68% of patients (Fig. 1b) and time from first infection to examination ranged from 3 to 42 months (Fig. 1c). Ongoing POS duration upon examination was at least one year in 78.2%, and at least 2 years in 33.3% of subjects (Fig. 1d). In addition, 33% in the POS group were on part-time or full-time sick leave from work, of which only 39% had a formal long-COVID diagnosis.

### Visual function and quality of life questionnaire

Rasch analysis of the modified Catquest-9SF survey responses indicated good psychometric properties and precision, with 2.76 logits person separation, reliability 0.88, and 4.40 strata (distinguishable levels of respondent ability/perception of difficulty). Item (question) separation was 7.89 logits (reliability 0.98, 7.78 strata), indicating the ability to reliably capture multiple strata of visual function without repetition. The POS group had significantly higher vision disability compared to CO (−0.65 ± 2.26 vs −5.44 ± 2.00 logits, mean ± SD, *P* < 0.0001), indicating strong perceived deterioration of vision in daily life, highly skewed towards disability (Fig. 1e). Responses indicated difficulties in daily life because of vision (item A, −1.57 ± 0.17 logits; mean ± SE) and dissatisfaction with vision (item B, −2.38 ± 0.18 logits), with tasks of reading text in a newspaper (item C1, −0.5 ± 0.16 logits) and from a screen (item C6, −1.08 ± 0.16 logits) being the most difficult (Fig. 1f).

Free-text self-described symptoms were identified as 22 distinct symptoms, with 75% of responders reporting 2–5 symptoms concurrently (Fig. 1g). Light sensitivity/photophobia (42.7%), eye pain (30.2%), eye fatigue (26.0%) and reduced focusing ability (22.9%) were the most frequent, followed by a range of ocular and neurological symptoms (Fig. 1h). The frequency of symptoms reported by females and males is indicated separately; however, no significant difference in the prevalence of symptoms was noted between females and males (minimum FDR = 0.38).

### Clinical examination

**Fig. 2:**
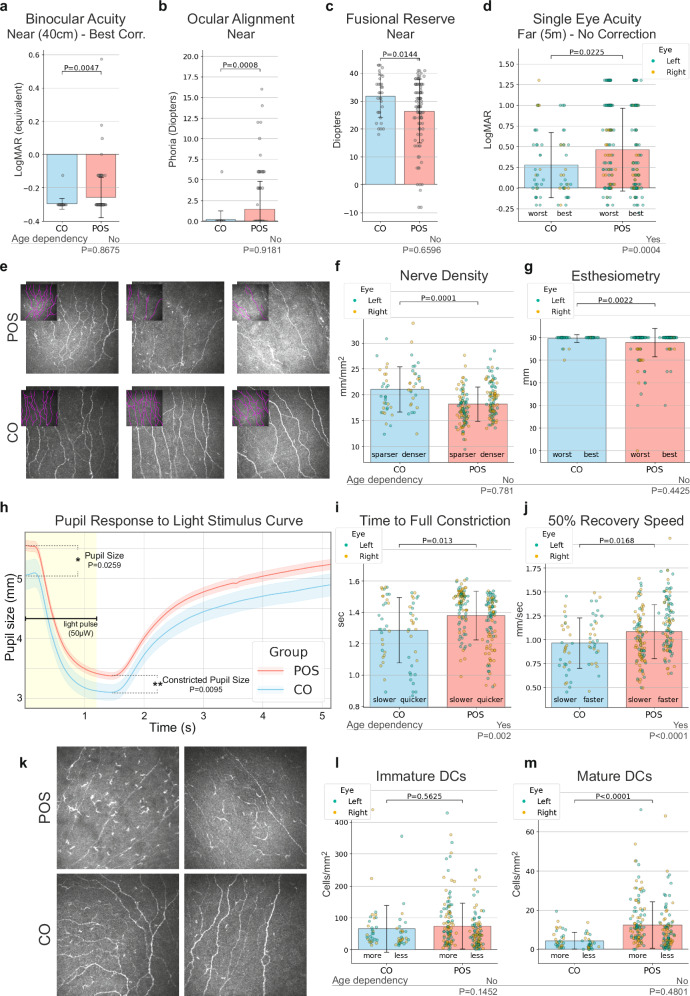
Specialized ophthalmic examinations reveal diminished ocular function and neuroinflammatory changes in POS. **a**–**c** Measures of near ocular vision and function and **d** uncorrected distance visual acuity with corresponding *P* values (age-adjusted where appropriate). **e** Corneal subbasal nerve live imaging (insets illustrate nerve tracing) and **f** corresponding subbasal nerve density quantification expressed in mm total nerve length per mm^2^ area of the central corneal plexus along with **g** functional corneal nerve testing for blink response by esthesiometry. **h** Dynamic pupil response curve to light stimulus indicating pulse duration, initial dark-adapted pupil size, and minimum constricted pupil size, with curves depicted as the group means (solid lines) with standard error (surrounding thick lines). **i** Time to full pupil constriction and **j** speed of recovery to 50% of the initial pupil size. Pupil parameter definitions are shown graphically in Supplementary Fig. 2a. **k** Live imaging of the corneal nerve plexus depicted dendritic/T cells (DCs) with **l** cell density quantified in morphologically distinct immature (non-antigen-presenting), and **m** mature (antigen-presenting) subsets (exact *P* value: 2.49 × 10^−9^). All bar graphs present data as mean values ±  standard deviation, overlaid with single data points per eye/participant. In parameters where two eyes were measured per participant, the group-wise statistical test was performed on the participant as the biological replicate. Source data for panels abcdfghijlm are provided as separate .csv files in the Source Data container file. Group sizes (*n*) and numerical values of graphed means, minimum, and maximum values are provided in the Graph_data_summary file in the Source Data container file. All statistical tests are two-sided, and were performed under the same scheme of testing for equality of variances and age confounding, as described in “Methods”. Graphed *P* values are unadjusted for multiple comparisons. POS persistent ocular symptoms group, CO control group, LogMAR logarithm of the minimum angle of resolution; *P < 0.05; **P < 0.001.

Standard anterior eye examination and refraction tests in POS patients did not reveal changes in uncorrected or distance-best-corrected visual acuity, distance ocular alignment/fixation, distance fusional buffer/reserve, refractive errors, corneal thickness, corneal endothelial cell density, tear film production (Schirmer test and tear meniscus), tear film quality (break-up time test), ocular bulbar redness, near point of accommodation, or stereoacuity (Supplementary Fig. 1). Measurements of near vision, however, revealed deficits in best-corrected binocular near visual acuity (mean difference POS-CO, +0.038 logMAR, *P* = 0.0047) and near ocular alignment/fixation ( + 1.29D, *P* = 0.0008), both testing coordinated eye movement to sharply focus on near objects and indicating a latent misalignment (inward turning, esophoria) of the eyes (Fig. 2a, b). The POS group also had a depleted near vision fusional reserve (−5.38D, *P* = 0.014; Fig. 2c), a measure of functional reserves to correct for ocular misalignment, and poorer uncorrected monocular distance visual acuity ( + 0.19 logMAR, *P* = 0.022; Fig. 2d).

Specialized clinical imaging and functional tests revealed further differences. In vivo confocal microscopy (IVCM) of the corneal subbasal nerve plexus with stringent dual-observer image selection and independent repeated analysis criteria indicated reduced subbasal epithelial nerve density in the POS group (−2.85 mm/mm^2^, *P* = 0.0001; Fig. 2e, f) while central corneal esthesiometry revealed a weakened blink reflex (−1.79 mm, *P* = 0.0022, Fig. 2g). The autonomic pupil reflex, quantified by dynamic pupillometry, indicated an upward-shifted response curve in the POS group (Fig. 2h), with larger dilated and constricted pupil size ( + 0.47 mm, *P* = 0.026 and +0.29 mm, *P* = 0.0095, respectively; Fig. 2h and Supplementary Fig. 2a–c). Additionally, we found a weaker constriction response evidenced by longer constriction time ( + 0.09 s, *P* = 0.013; Fig. 2i) and faster velocity to 50% recovery of dilation ( + 0.12 mm/s, *P* = 0.017; Fig. 2j). Imaging and quantification of inflammatory dendritic and T cells (DC/T) in the subbasal nerve plexus by IVCM^9^ (Fig. 2k) revealed no change in immature DC/T density (non-antigen-presenting, +8.03 cells/mm^2^, *P* = 0.56; Fig. 2l), but elevated mature DC/T density (activated or antigen-presenting; +8.05 cells/mm^2^, *P* < 0.0001; Fig. 2m) in POS.

### Tear proteomics

**Fig. 3:**
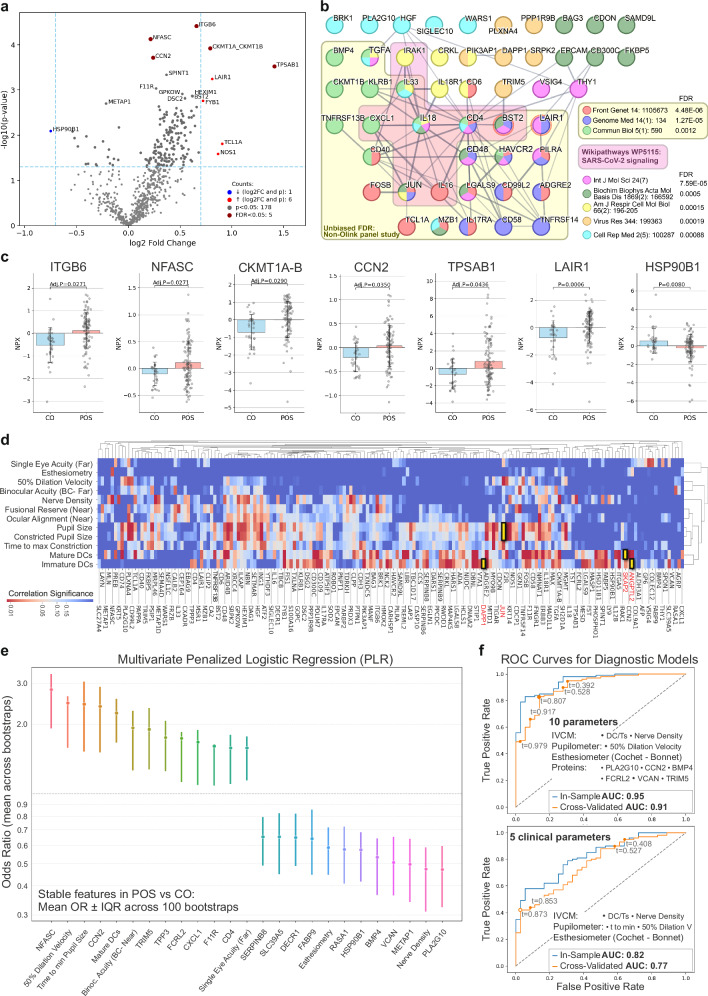
Tear film proteome reveals severe COVID-19 signature and identifies putative neuroinflammation biomarkers with high clinical correlation and diagnostic utility. **a** Volcano plot indicating 178 dysregulated tear proteins (dark gray dots, unadjusted *P* < 0.05) in the persistent ocular symptoms group (POS) relative to the control group (CO), of which 6 had log_2_ fold change (log_2_FC)>0.7 (red and dark red dots in top right), and 1 had log_2_FC < -0.7 (blue dot). Five proteins had adjusted false discovery rate (FDR) < 0.05 (dark red dots). **b** STRING network overlap of dysregulated tear proteins with published COVID-19 blood and tissue targets from unbiased -omics data (non-Olink platforms, yellow bubble), with known targets from the SARS-CoV-2 signaling Wikipathway (pink bubble), and with five COVID-19 cohorts using the Olink platform (no bubble). FDR is for the statistical significance of the overlap with the respective cohort, and targets (labeled circles) are color-coded according to overlapping cohort (divided circles indicate multiple overlapping cohorts). **c** Distribution of top dysregulated protein levels across groups (one circle/participant, over bar graph of mean values ± standard deviation. Adj.p: Benjamini–Hochberg FDR 0.05 adjusted *P* value. Other graphed *P* values are unadjusted for multiple comparisons. **d** Hierarchical clustering heatmap of Pearson correlation significance between clinical ocular parameters and dysregulated tear proteins. Yellow rectangles indicate significance after FDR adjustment. **e** Odds ratio (OR) and interquartile range (IQR) for clinical parameters and proteins with the greatest discriminating power for POS in a multivariate penalized logistic regression model across 100 bootstraps. Numeric descriptions of all mean values and IQR whiskers in the graph are provided as a .csv file in the Source Data container file. **f** Receiver-operating characteristic (ROC) curve for optimized diagnostic models for POS with 4 clinical parameters plus 6 tear proteins (top) and with only clinical parameters (5 parameters from 3 instruments) (bottom), indicating area under the curve (AUC) for model classification in-sample (blue curve) and after fivefold cross-validation (orange curve). Various threshold diagnostic values (*t* values) are indicated. Model equations and thresholds are provided in Supplementary Table 3. Source data for (**c**, **d**) are provided as separate .csv files in the Source Data container file. Group sizes (*n*) and numerical values of graphed means, minimum, and maximum values are provided in the Graph_data_summary file in the Source Data container file. All statistical tests are two-sided, and were performed under the same scheme of testing for equality of variances and age confounding, described in “Methods”. NPX normalized protein expression level (Olink panel output units), BC best-corrected binocular acuity, DC/Ts dendritic cells/T cells, IVCM in vivo confocal microscopy.

Tear fluid analyzed by targeted proteomics for 768 proteins by Olink proximity extension assay revealed 178 dysregulated proteins (*P* < 0.05) in POS, of which 6 had log_2_ fold change (log_2_FC)>0.7 and 1 had log_2_FC < −0.7 (Fig. 3a). STRING network analysis of the dysregulated proteins (Supplementary Fig. 3) highlighted the adaptive immunity and TNF ligand-receptor binding cluster, central to which was CD4, and the HSP90 chaperone cycle^10^. The dysregulated tear protein network overlapped significantly with blood transcriptome and DNA methylome targets from three published cohorts with severe COVID-19^11–13^ and overlapped with the SARS-CoV-2 signaling WikiPathway (WP5115). Further overlap included dysregulated blood and tissue proteins from five COVID-19 cohorts (severe, fatal, and long-COVID) using Olink platforms^14–18^ (Fig. 3b).

Of the dysregulated tear film proteins, five had significant FDR (< 0.05) after multiple comparison adjustment: integrin-β6 (ITGB6), neurofascin (NFASC), mitochondrial creatine kinase 1A/1B (CKMT1A-CKMT1B), the connective tissue mitoattractant CCN2 (also known as CTGF), and Tryptase α/β-1 (TPSAB1) (Fig. 3a, c). Additional proteins with high significance and |log_2_FC| were LAIR1, and the downregulated HSP90B1 (Fig. 3a, c). Group means, log_2_FC, *P* values, and FDR-adjusted *P* values are listed in Supplementary Table 2. Cross-correlation between dysregulated proteins and clinical measurements (Fig. 3d) revealed significant correlations of dilated and constricted pupil size with JUN (FDR = 0.004 and 0.005, respectively), of immature DC/T with ANGPTL2 (FDR = 0.007) and DAPP1 (FDR = 0.02), and of mature DC/T with SKAP2 (FDR = 0.02). Symptom-based binary classification revealed associations between weakened pupillary constriction and self-reported light sensitivity, reduced focusing adjustment, moving text perception, and headaches. Monocular distance acuity was associated with self-reported reduced focusing ability (Supplementary Fig. 4).

### Diagnostic models

Given the current lack of objective diagnostic criteria for POS, we performed a multivariate penalized logistic regression analysis utilizing clinical parameters and tear protein levels to distinguish POS from CO in individual subjects. The most stable and highly discriminatory model parameters are shown in Fig. 3e. Because tear film diagnostics are not yet part of standard clinical testing, a first diagnostic model was constructed using five clinical parameters (requiring three instruments) yielding a cross-validated 77% AUC accuracy (Fig. 3f). Four parameters measure neural activity (subbasal nerve density, blink response, constriction time and dilation velocity) and one measures inflammation (mature DC/T density). For improved accuracy, this model was augmented with 6 tear protein levels, yielding a second model with 91% cross-validated AUC (Fig. 3f). All model coefficients are provided in Supplementary Table 3. Unbiased hierarchical clustering of study subjects based on proteomic results revealed a clear group-wise separation (Supplementary Fig. 5a). Broad tear proteome perturbation was associated with symptoms of reduced focusing ability and focus adjustment, headache behind the eyes, and multiple/singular SARS-CoV-2 infections (Supplementary Fig. 5b).

## Discussion

With cases of COVID-19 not requiring hospitalization being ubiquitous, a potentially large population may develop ocular symptoms, as in the POS group, that can be undiagnosed and difficult for ophthalmologists or neurologists to assess on an individual basis. Importantly, although most post-COVID-19 sequelae subside at 2 years post-infection^19^, symptoms in our cohort were persistent and active during all study examinations, with duration up to 3 years in some subjects. The high level of disability and characteristic symptoms may partially be explained by ocular misalignment, depleted fusional reserves, and reduced near vision and monocular acuity, all components of strabismus, leading to classic symptoms of headaches, eye fatigue, blurred vision, double vision, and ocular discomfort^20^. When resolving small details, visual acuity is sensitive to suboptimal pupil size^21^ and in our data, a pupillary dysautonomia with weakened pupil constriction responses correlated with reported light sensitivity, headaches, reduced focusing ability, and reduced focus adjustment. Weakened blink reflex and reduced density of corneal subbasal nerves were further noted while concurrently, in the corneal subbasal nerve plexus, the increased activation of DC/T cells suggests an inflammation-mediated nerve degeneration in POS. This is supported by reports of corneal nerve fiber loss and increased DC density at least 20 months after COVID-19^22,23^ and small fiber neuropathy in the cornea up to 12 weeks after mild SARS-CoV-2 infection^24^. Our findings are consistent with corneal neuralgia in POS, a debilitating condition of ocular pain, irritation, burning, and photophobia, notoriously difficult to diagnose^25,26^. Notably, in a study examining PBMCs from severe COVID-19 patients, elevated DC subsets persisted 6–7 months following infection^27^. The elevated DC/T in POS is consistent with the dysregulation of the cluster of CD4, CD40, CD48, IL-16, IL-17RA, IL-18/R1, IL-33, TNFRSF13B and TNFRSF14 that we found in the tear fluid. This overlapped with PBMC expression profiles in multiple severe COVID-19 cohorts^11–17^, underscoring the long-term effects of SARS-CoV-2 on chronic T cell regulatory activity in the eye.

The most dysregulated tear film proteins in POS were ITGB6, NFASC, CKMT1A/B, CCN2, and TPSAB1. The first of these, ITGB6, is reported as a marker and exacerbating agent of COVID-19-induced tissue injury and severity^28^ and is a major driver of TGF-β-regulated T cell responses^29,30^. It has also been found as a wound healing marker in the cornea^31^, aligned with the chronic ocular surface inflammation and nerve loss we detected in POS. A central component of corneal inflammation is the recruitment, priming, and activation of DC/T cells that significantly correlated with upregulation of the key priming protein ANGPTL2^32,33^. Simultaneously, the dysregulation of SKAP2 and DAPP1 correlated with, and highlighted DC-mediated T cell maturation as a prime event in POS corneal inflammation^34,35^.

The upregulation of NFASC and the diminished corneal nerve density, blink reflex, and pupillary response in POS are consistent with sustained neuropathy. NFASC, reported to be upregulated in severe long-COVID with cognitive impairment^36^, is a regulator of neuronal growth^37^, linked with chronic inflammatory demyelinating polyneuropathy^38^, autoimmune neuropathies^39^, and optic nerve demyelination^40^. The correlation of increased pupil size with elevated JUN levels, a major transcription factor in the peripheral nerve injury response in various sensorimotor neuropathies^41,42^, indicates a plausible role in autonomic pupillary response impairment and, like NFASC, suggests demyelination, with peripheral nerve injury triggering JUN expression to orchestrate neural repair^43^ and demyelination^44^. The significant dysregulation of several other proteins like the mitochondrial creatine kinase CKMT1A/B, TPSAB1, and LAIR1, the latter two previously reported as markers of severe COVID-19^45,46^, suggest a mitochondrial oxidative stress response in neural cells,^47^ an autonomic nerve dysfunction^48^, inflammation, and immune dysregulation^49,50^. Importantly, given the strong crosstalk and reciprocal relationship of DC/T cells and subbasal nerves in the corneal epithelium^51,52^ with mature corneal DCs representing MHC class II antigen presenting cells^53^, our findings of neuropathy and mature DC/T cell elevation in the cornea in POS are consistent with a COVID-induced chronic neuroinflammation. Notably, our tissue and protein-level findings of chronic T cell dysregulation in the eye are consistent with results of systemic T cell dysregulation in blood from mild-to-moderate^54^ and long COVID^55^ populations and sustained aberrant T cell activation in long COVID-associated neurological cognitive impairment^56^. Although we did not include a control group without prior SARS-CoV-2 infection, as we could not definitively establish the absence of asymptomatic COVID-19 or absence of infection-related SARS-CoV-2 antibodies in a broadly vaccinated population, we compared results with pre-pandemic normative data for corneal subbasal nerves^57–59^, mature DC/T cells^60,61^, and central corneal esthesiometry^62^. Normative data in healthy individuals revealed values at the same levels as in our control group, indicating the neural and inflammatory changes observed are not normally present in a healthy population, and are evident in POS but not following COVID-19 generally.

Limitations of our study are its size and lack of overall prevalence estimates for POS following mild SARS-CoV-2 infection. With post hoc power testing, however, at an effect size of *d* = 0.6, 91% of parameters achieved ≥ 80% power, and all parameters achieved ≥ 78.4% power, indicating the study was sufficiently powered to detect moderate-to-large effects, whereas smaller effects would require larger populations to detect. Also, we relied on self-reported symptoms, which provided limited data on concurrent long-COVID, and we did not acquire detailed data on symptom severity, viral variants, or type and frequency of vaccination. Study design precluded examination in relation to singular infection events; patients typically reported symptoms worsening with subsequent infections. We did not examine blood or other body fluids; however, our proteomic results are specific to the eye, strengthening our functional and morphological findings and their linkage to POS. As we did not perform ocular biopsies, however, it remains to be established whether the DC/T subsets observed are directly expressing the detected T cell proteins or mirroring systemic expression as indicated in the tear film layer.

In conclusion, we report and formally describe a set of persistent, quality-of-life-affecting and debilitating ocular symptoms arising after recovery from mild COVID-19 that did not require hospitalization. We also identify underlying pathologies arising secondary to infection (strabismus, pupillary dysautonomia, peripheral corneal neuropathy, and neuropathic ocular pain) and possible diagnostic methods and biomarkers. Based on our findings, we believe further studies are needed to confirm an undiagnosed population with a high burden of disability from ocular sequelae we consider as a post COVID eye syndrome of neuroinflammatory origin.

## Methods

### Study design and participant recruitment

A prospective observational study was conducted including 100 participants experiencing symptoms related to the eyes or vision after, but not prior to mild SARS-CoV-2 infection, and 32 controls without ocular symptoms following mild SARS-CoV-2 infection. Subjects from the general population with specific ocular health concerns after COVID-19 contacted the study investigators for possible inclusion in the study following broad Swedish media reporting (newspaper, radio, television, and internet) of the proposed study. Subjects were subsequently screened for confirmed COVID-19 diagnosis based on positive qPCR test result, or during the period of Feb-May 2020 when tests were not widely available in Sweden, a strong suspicion of COVID-19 infection based on acute symptoms (fever, loss of smell or taste, fatigue, headache, sore throat, nasal congestion) and presence of risk factors (such as travel to region with reported outbreak or symptomatic infection within the household). Medical records and screening questions were used to confirm the absence of prior ocular diagnoses, ocular surgeries, or chronic diseases with possible ocular manifestation including diabetes and other systemic autoimmune or neurodegenerative diseases. Inclusion criteria were: aged 4 years and older (for compliance with testing procedures), had recovered from mild COVID-19 without hospitalization, and debut of visual or ocular symptoms persisting after infection and with a duration of at least 12 weeks at the time of examination. Subjects under the age of 18 were included only after signed informed consent was obtained from all legal guardians. Exclusion criteria were history of prior ocular surgery or eye disease, severe ocular comorbidities not related to the anterior eye (such as age-related macular degeneration, retinal detachment, optic nerve pathology, etc.), eye deformities, and a history of daily contact lens wear (past short-term or intermittent contact lens usage in the past, for example, during sporting activities, was however, allowed). 32 age-matched volunteers with prior SARS-CoV-2 infection but no prior or current ocular symptoms or systemic comorbidities were recruited and served as controls. All participants provided voluntary written signed informed consent to participate in the study. The study was initiated following approval by the Swedish Ethical Review Authority (approval nos. 2022-00365-01 and 2022-04607-02) and adhered to the tenets of the Declaration of Helsinki, October 2013.

### Questionnaires and patient history

Participants completed a Modified Catquest-9SF Questionnaire prior to the study visit. This standard validated questionnaire, adapted to the Swedish language, was used to evaluate visual function and quality of life. The Catquest-9SF contains nine items, all of which form a scale to measure a unique underlying trait—the patient’s self-assessed visual disability and satisfaction with their vision (with higher values, in logit units, corresponding to higher disability)^63^. The items used are presented in Supplementary Table 4, with the modified item being item C6. The original version contains [Reading subtitles on TV], and the applied change was [Reading from screens] to better capture the difficulty in using screens generally. Responses were recorded on a standardized Likert scale and fitted to the Rasch model using WINSTEPS v4.0.1 and jMetrik. Prior to the study visit, participants provided detailed free-text descriptions of perceived ocular symptoms post-infection. Additional screening questions and structured data were collected during the study visit prior to clinical examinations, including timing of COVID-19 infection, infection severity, presence of systemic disease, comorbidities, history of ocular surgeries, use of ocular medications (e.g., eye drops), and usage of contact lenses. Text mining of the written free-text symptom descriptions was performed to extract commonly reported symptoms that were subsequently aggregated into meaningful symptom categories. Participants were in some cases contacted via email or telephone following the study visit to confirm and/or receive additional data regarding dates of COVID-19 infection and ocular symptom onset.

### Clinical ophthalmic examinations

During a single visit to the Eye Clinic, Linköping University Hospital at the Department of Biomedical and Clinical Sciences, Linköping University, with all visits conducted between April 2022 and September 2023 and all tests performed by the same investigators, each participant underwent a comprehensive ophthalmic clinical examination conducted by trained ophthalmic professionals, including the following tests. Visual acuity was measured uncorrected and with best-spectacle refractive correction for distance vision using a 5-m decimal Snellen chart and for near vision using the T-scale at 40 cm, done monocularly (each eye) and binocularly (both eyes). Visual acuity measurements were converted to LogMAR values for statistics and reporting. Visual acuity was measured with patients’ current refractive correction (presenting visual acuity), after which autorefraction was performed with the Topcon KR8900 Auto Kerato-Refractometer. Subjective refraction was performed to find the best possible refractive correction producing the best-corrected visual acuity. Binocular vision and strabismus were measured with the Bagolini Striated Glasses test for sensory fusion, TNO Stereo test for stereo acuity (cards for balls, butterfly, and from 480 up to 60 arcseconds, converted for statistics to log_2_ arcseconds), assessment of accommodation amplitude (Royal Air Force Rule, in diopters), and examination of ocular motility and alignment including detection of strabismus (cover test), vergence and convergence function evaluations (fusional width, fusional reserve).

Ocular surface assessments were performed using the Oculus Keratograph 5 (Oculus GmbH). We measured bulbar and limbal conjunctival redness (average of three consecutive measurements per eye), Non-Invasive Keratograph Break-Up Time (NIKBUT; average of three consecutive measurements per eye), and tear meniscus height (average of three consecutive measurements per eye). Tear film production was quantitatively measured over a 5-minute period using the Schirmer test without anesthesia and reported in millimeters from the paper test strips. The test strips, impregnated with tear fluid (test strips without ink markings, Haag-Streit UK Ltd.), were saved for subsequent tear film analysis. Pupillary function was objectively assessed using dynamic pupillometry (NeurOptics PLR-3000 pupillometer). In a darkened room, pupillary responses to a standardized 50 μW 1.2 s flash stimulus were recorded by image recognition of the pupil in 30 fps images over 6 s. From the recorded pupil size data, parameters were measured and calculated including initial pupil size, constricted pupil size, time to maximum constriction, constriction velocity, 50% dilation recovery time and dilation velocity (Supplementary Fig. 2a). Measurements for all subjects were conducted by the same operator; each measurement was repeated three times per eye, and data were averaged separately per eye.

Corneal endothelial cell density was evaluated by noncontact specular microscopy (Topcon SP3000P specular microscope), averaging the count of three measurements of endothelial cells for each participant eye, by automated cell count analysis using the built-in software. Corneal surface mechanical sensitivity was quantitatively measured using a Cochet–Bonnet handheld esthesiometer to determine the blink reflex threshold, expressed in mm of nylon thread length for blink stimulation. Measurements were performed three times centrally per eye, with data averaged separately for each eye. Central corneal thickness was evaluated using optical coherence tomography imaging (Optovue iVue OCT, Optovue Inc.). Each eye was scanned three times, and an average corneal thickness was calculated based on a 2 mm diameter central corneal region.

Corneal nerve and inflammatory cell imaging were conducted using in vivo confocal microscopy (IVCM) with the HRT3-RCM system (Heidelberg Retinal Tomograph 3 with Rostock Cornea Module, Heidelberg Engineering, Germany). Multiple sets of captured image sequences covering the anesthetized central cornea were taken for all subjects by an experienced operator, as the final clinical examination. For corneal nerves, each of two independent trained and masked observers, experienced in IVCM image analysis, selected five representative images from standardized locations near and superior to the nerve plexus spiral center, where subbasal nerves exhibited a predominantly vertical orientation, without image overlap. Observers analyzed their own selected images independently, tracing nerve fibers semi-automatically using ImageJ/Fiji software^64^ with the NeuronJ plugin^65^. Nerve length was measured and expressed as an average total nerve length per unit area of the corneal subbasal nerve plexus. A separate set of five representative images per eye was chosen independently by each observer using the anatomic nerve orientation described above as a landmark (after reviewing the entire captured set of images), and was used for inflammatory cell quantification from the central subbasal nerve plexus using the ImageJ cell counter plugin. Immature (reflective elongated cell bodies without or with short dendrites <25 µm in length) and mature dendritic cells (reflective cell bodies with multiple long dendrites and/or dendrites ≥25 µm in length) were marked separately based on morphology^60,66^ and counted in each of the selected images, and an average cell count per unit area was calculated.

### Tear film proteomics

Tear samples collected from the Schirmer test in the form of wet paper strips from both eyes were stored individually in cryotubes and snap-frozen by immersion in dry ice-cold isopentane, then kept on dry ice until storage and for a maximum of 3 hours. Samples were stored at −80 °C in the Linköping University Biobank prior to downstream analysis. Samples from a single randomly chosen eye of each participant were utilized for extraction and analysis (one distinct sample from each eye of each individual). Proteomic analysis of tear samples was conducted using the Olink Explore 384 Inflammation I and Explore 384 Neurology I panels. Processing involved eluting each sample by immersing the frozen Schirmer paper strips in Costar Spin-X filter tubes with 300 μL PBS 0.05% Tween-20 1% BSA and incubating for 10 min in RT above the filter, then centrifuging in a 4 °C cooled microcentrifuge at 13,000×*g* for 10 min and keeping the eluate while discarding the filter. In total, 40 μL per sample (plate well) were immediately plated into Olink 96-well plates in programmatically randomized plate positions. Subsequent analysis was performed according to the standardized Olink 384 protocol procedures at the SciLifeLab core facility in Uppsala, Sweden. All significant (unadjusted *P* value) protein targets between CO and POS were input into the STRING platform for pathway analysis. Although highly significant, most pathway FDR values reported by the tool were disregarded as biased, because the tool assumes a target list that originates from an unbiased source of potential hits, while the nature of the panels we used was specific to inflammatory and neurological processes, thus enriching those pathways. However, we considered correlation with other published datasets as valid, when those publications did not utilize the Olink platform as the source of high-throughput data. Also, we considered unbiased clustering and correlations between significant targets as valid, and we reported the subset of our hits that are described as part of SARS-CoV-2 signaling in Wikipathways.

For a limited independent validation of the most significant targets, we utilized tear samples from the contralateral eye in 7 CO and 6 POS randomly chosen participants, after filtering participants based on our 10-parameter outcome predictor model (see below) using Youden’s J as a threshold (CO below the threshold, POS above), and performed Western blot analysis. Validation was performed for proteins ITGB6 (Origene mouse monoclonal anti- ITGB6, clone OTI1D2, Catalog # TA507354S, dilution 1:500), NFASC (Origene mouse monoclonal anti-Neurofascin, clone OTI11A7, catalog # TA815269S, dilution 1:500) and CCN2 (Bio-Rad mouse monoclonal anti-CTGF, catalog # VMA00685, clone AB01/1E5, dilution 1:500). For detection we used a goat anti-mouse IgG (H + L) Poly-HRP secondary antibody (Invitrogen by ThermoFisher, ref 32230, dilution 1:2000). Quantification of total protein and band density was performed with the Image Lab software (v6.1, Bio-Rad). Validation data are presented in Supplementary Fig. 6.

### Data analysis—statistics

For comparison of symptom frequencies between female and male participants where a symptom was reported six times or more (*n* = 18), a chi-square test was performed, and resulting *P* values were adjusted for multiple comparisons using the Benjamini–Hochberg false discovery rate (FDR) method. For each clinical parameter and protein measurement in tears, differences between control (CO) and experimental (POS) groups were assessed while accounting for age as a potential covariate. Significance was defined as a two-tailed *P* value < 0.05. Sample sizes for each group were calculated per parameter, accounting for any missing values (Supplementary Table 5). Power analyses were performed post hoc for each study parameter using observed sample sizes and variability. For each, we report the minimum detectable effect size (MDES, Cohen’s *d*) and simulated power for *d* = 0.6 (Supplementary Table 5). All analyses were performed using Python (pandas, statsmodels, and scipy libraries), and results were exported for further reporting. Analyses were conducted using multiple linear regression models with and without age as a predictor (ANCOVA-style), treating group (CO vs. POS) as a categorical independent variable and age as a continuous covariate. Prior to regression analysis, we tested for equality of variances between groups using Levene’s test. If the test indicated no significant difference in variances (*P* > 0.05) for the specific parameter, standard ordinary least squares (OLS) regression was used. If variances were unequal (*P *≤ 0.05), we applied OLS regression with heteroscedasticity-consistent standard errors (HC3 estimator) to account for unequal variance.

Under equal variance assumptions, two model comparisons were conducted to evaluate whether including age significantly improved model fit. This was done using an F-test comparing the full model (group + age) to the reduced model (group only). When variances were unequal, age model selection was based on the significance of the age coefficient in a model fit using heteroscedasticity-consistent (HC3) standard errors. Based on this comparison, the model with a better fit (*P* < 0.05) was selected as the preferred model for reporting the group effect. In measurements where it was meaningful to have distinct data from both eyes, we performed statistical analysis in 3 ways: worst eye measurement per participant, best eye measurement per participant, and both measurements treated as co-dependent variable pairs. For protein measurements in tears, FDR and multiple comparison-adjusted *P* values were calculated according to Benjamini–Hochberg using statsmodels.stats.multitest in Python.

### Correlation analysis—outcome predictor modeling

Pearson correlation *r*^2^ values, *P* values, and slopes between measured parameters that were significant in CO vs POS and the tear sample protein relative abundances were calculated for all parameter–protein pairs in Python using the scipy.stats library. Also, the ten most frequently reported symptoms were treated as binary outcomes to segregate study participants into groups of [symptom] vs [no symptom]. For all groupings, each clinical parameter and protein abundance level was tested with a *t* test (equal variances decided with a Levene’s test, similar to the CO vs POS comparisons). Multiple comparison-adjusted *P* values (Benjamini–Hochberg) were then computed.

To assess multivariate relationships and identify predictors of clinical outcomes, a penalized logistic regression (PLR) model using L1-regularized logistic regression (LASSO) was fitted to predict the binary CO vs POS outcome using the scikit-learn, seaborn, and matplotlib libraries. The model used all available clinical parameters and protein measurements as predictors. Data were standardized, and missing values were imputed using the column-wise mean. A separate model was then trained, and features with non-zero coefficients were considered predictive. The area under the receiver operating characteristic curve (AUC) was computed to assess discriminative performance. To evaluate the robustness of the feature selection and model accuracy in distinguishing POS from CO groups, we implemented a bootstrapped PLR approach where POS vs CO classification was modeled using logistic regression with L1 regularization. The modeling procedure was repeated 100 times on bootstrap-resampled subsets of the data (with stratified sampling to preserve class balance). For each bootstrap iteration, model coefficients and AUC values were recorded. For each predictor, the mean, standard deviation, and selection frequency (i.e., the proportion of bootstraps in which the feature had a non-zero coefficient) were calculated. Only features selected in >50% of bootstraps were considered, and the top 25 (mean OR furthest from 1) were retained for visualization and further consideration in model construction. Based on the results of the bootstrap feature-stability analysis, we next constructed diagnostic models using either only clinical parameters, or the clinical parameters in combination with subsets of protein measurements identified in the bootstrap screen. We exhaustively evaluated all possible combinations of the clinical features together with up to six additional protein markers drawn from the bootstrap-derived candidate set. For each candidate model, a PLR model (L1-regularized, fitted with the saga solver) was trained. Model discrimination was assessed by calculating the area under the receiver operating characteristic curve (AUC) in two ways: with an apparent estimate obtained by fitting and evaluating on the full dataset, and with an out-of-fold cross-validated estimate, obtained by repeated stratified fivefold cross-validation, where predictions for each held-out fold were aggregated to form the cross-validated ROC curve. The best-performing combinations were selected based on the highest cross-validated AUC values.

### Reporting summary

Further information on research design is available in the Nature Portfolio Reporting Summary linked to this article.

## Supplementary information

Supplementary Information

Peer Review file

Reporting Summary

## Source data

Source Data

## Data Availability

Source data for all figure panels are provided with this paper as per-panel .csv files in a zipped container Source Data file. Individual, anonymized source data and IVCM images have been deposited in the Figshare database under accession code 10.6084/m9.figshare.30245398. The Swedish Health Authority national statistics on COVID-19 were sourced and are also available at https://www.folkhalsomyndigheten.se/faktablad/fall-covid-19/. The Swedish-translated version of the Catquest-9SF survey and its layout design as presented to the participants is available upon request. All processed data generated in this study are provided in the Supplementary Information and the Source Data file. Source data are provided with this paper.
