# Supplementary material for: Long-term ocular symptoms following COVID-19 linked to immune dysregulation, dysautonomia and peripheral neuropathy
Source: Nat Commun. 2026 Jul 8;17:5624. doi: 10.1038/s41467-026-74858-4 (PMC13346420; doi:10.1038/s41467-026-74858-4)
Supplement: Supplementary file 1 — Supplementary Information [file 41467_2026_74858_MOESM1_ESM.pdf]

Supplementary Information file

Long-term ocular symptoms following COVID-19 linked to immune dysregulation, dysautonomia and peripheral neuropathy

Petros Moustardas[1], Helen Setterud[1,2], Helena Meijer[2], Gunnel Andersson[2], Jenny Roth[3], Ava Dashti[1], Björn Johansson[1,2], António Filipe Macedo[3,4], Neil Lagali[1,2,5*]

[1]Division of Ophthalmology, Department of Biomedical and Clinical Sciences, Faculty of Medicine, Linköping University, 581 83 Linköping, Sweden

[2]Clinical department of Ophthalmology, Region Östergötland, 581 85 Linköping, Sweden

[3]Department of Medicine and Optometry, Linnaeus University, 39182 Kalmar, Sweden

[4] Department and Centre of Physics-Optometry and Vision Science, University of Minho, Braga, Portugal

[5]Department of Ophthalmology, Sørlandet Hospital Arendal, Arendal, Norway

[*]Correspondence: neil.lagali@liu.se

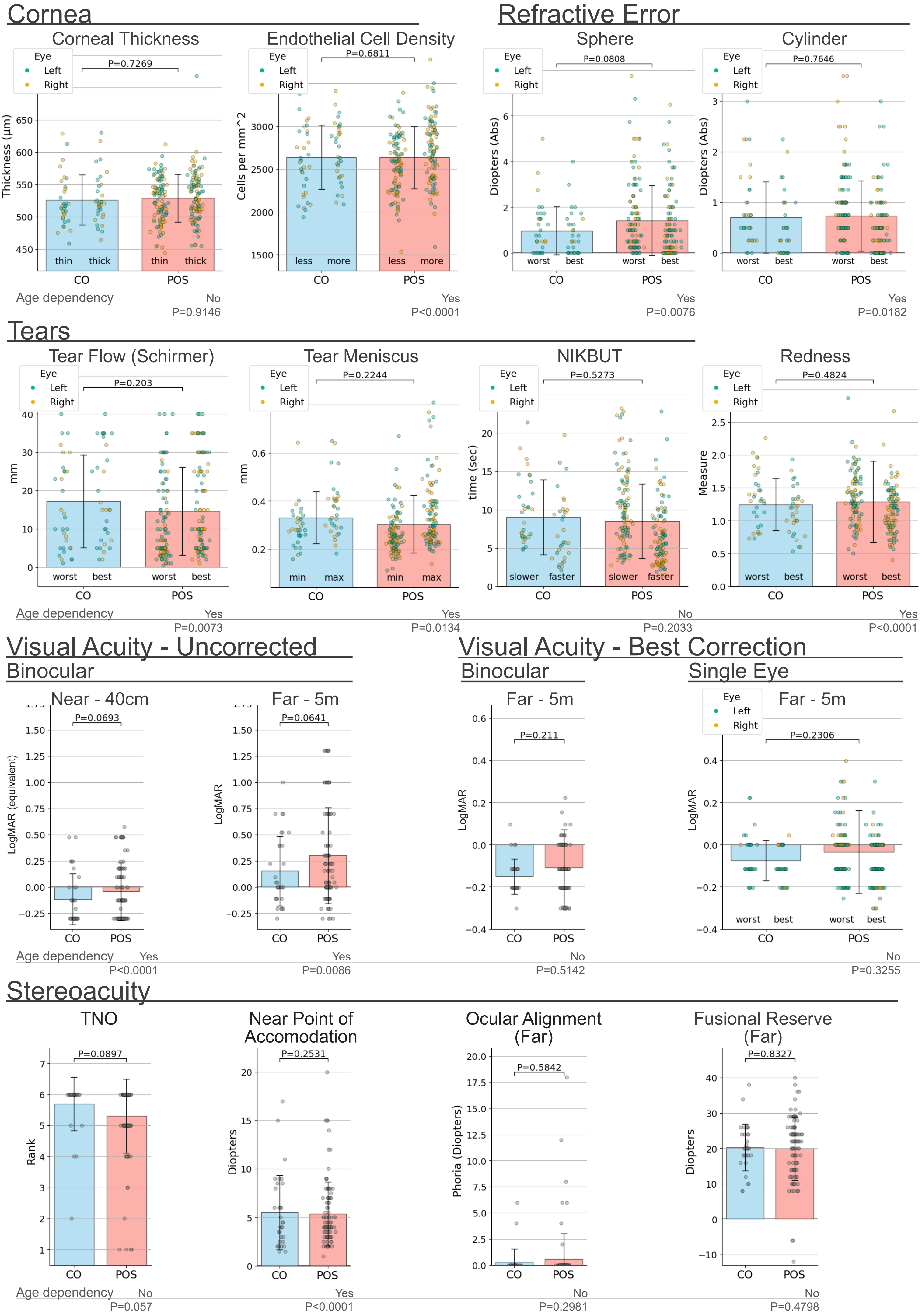
Cornea
Corneal Thickness
Eye
Left
Right
P=0.7269
Thickness (µm)
thin
thick
CO
POS
Endothelial Cell Density
P=0.6811
Cells per mm^2
less
more
Refractive Error
Sphere
P=0.0808
Diopters (Abs)
worst
best
Cylinder
P=0.7646
Age dependency
No
P=0.9146
Yes
P<0.0001
Yes
P=0.0076
Yes
P=0.0182
Tears
Tear Flow (Schirmer)
P=0.203
mm
Tear Meniscus
P=0.2244
min
max
NIKBUT
P=0.5273
time (sec)
slower
faster
Redness
P=0.4824
Measure
Age dependency
Yes
P=0.0073
Yes
P=0.0134
No
P=0.2033
Yes
P<0.0001
Visual Acuity - Uncorrected
Binocular
Near - 40cm
P=0.0693
LogMAR (equivalent)
Far - 5m
P=0.0641
LogMAR
Visual Acuity - Best Correction
Binocular
Far - 5m
P=0.211
Single Eye
Far - 5m
P=0.2306
Age dependency
Yes
P<0.0001
Yes
P=0.0086
No
P=0.5142
No
P=0.3255
Stereoacuity
TNO
P=0.0897
Rank
Near Point of Accomodation
P=0.2531
Diopters
Ocular Alignment (Far)
P=0.5842
Phoria (Diopters)
Fusional Reserve (Far)
P=0.8327
Diopters
Age dependency
No
P=0.057
Yes
P<0.0001
No
P=0.2981
No
P=0.4798

Supplementary Fig. 1 | Standard anterior segment examinations did not reveal changes in functional ophthalmic parameters in the post-COVID-19 persistent ocular symptoms (POS) group. Central corneal thickness, endothelial cell density, corneal refractive errors, tear film parameters, ocular bulbar redness, most measures of visual acuity and stereoacuity did not differ between POS and Control (CO) groups. All bar graphs present data as mean values ± Standard Deviation, overlaid with single data points per eye / participant. In parameters where two eyes were measured per participant, the groupwise statistical test was performed on the participant as the biological replicate. Source data, group sizes (n) and numerical values of graphed means, minimum and maximum values are provided in the Graph_data_summary file and in the All_clinical_measurements file in the Source Data container file. All statistical tests are two-sided, and were performed under the same scheme of testing for equality of variances and age confounding, described in the Methods section. NIKBUT: non-invasive keratograph break-up time; TNO: TNO stereoacuity (stereopsis) test, named after the Dutch organization where the test was developed; LogMAR: logarithm of the minimum angle of resolution.

Pupilometry

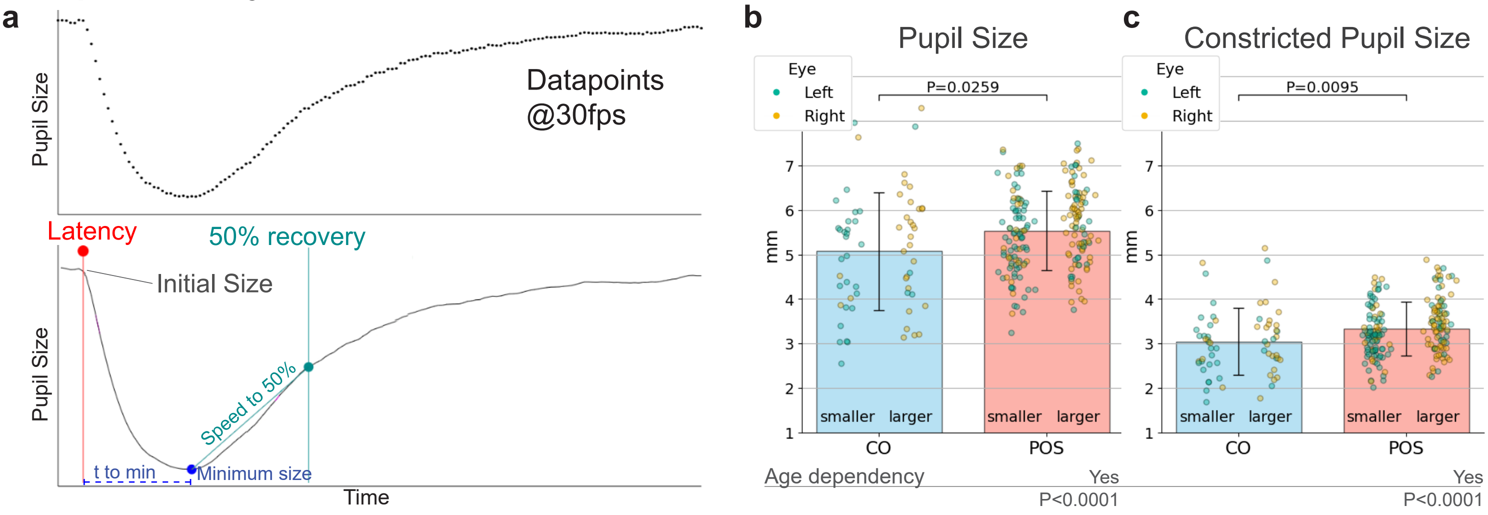

Supplementary Fig. 2 | Reduced pupillary light inhibition in the post-COVID-19 persistent ocular symptoms (POS) group. (**a**, upper) Typical data obtained from a dynamic pupil scan at 30 frames/sec and (**a**, lower) definition of measured pupil parameters. (**b**) Initial dark-adapted pupil size, and (**c**) minimum constricted pupil size. Source data, group sizes (n) and numerical values of graphed means, minimum and maximum values are provided in the Graph_data_summary file and in the All_clinical_measurements file in the Source Data container file. All statistical tests are two-sided, and were performed under the same scheme of testing for equality of variances and age confounding, described in the Methods section. POS: persistent ocular symptoms group; CO: control group; t: time; fps: frames per second.

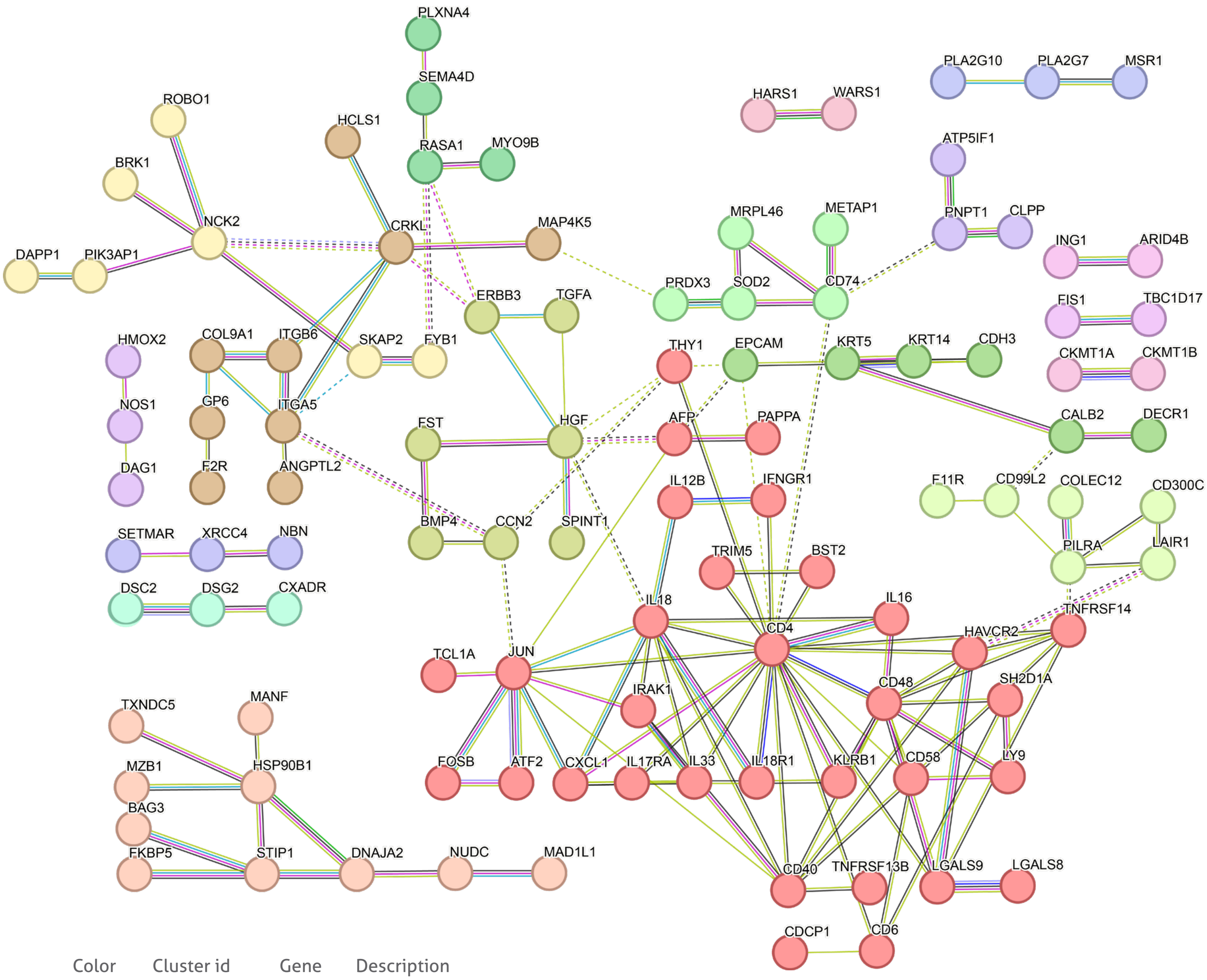

Color	Cluster id	Gene Count	Description
	Cluster 1	32	Mixed, incl. Adaptive immunity, and TNFs bind their physiological receptors
	Cluster 2	10	HSP90 chaperone cycle for steroid hormone receptors (SHR) in the presence of ligand
	Cluster 3	9	-ANGPTL2, COL9A1, CRKL, F2R, GP6, HCLS1, ITGA5, ITGB6, MAP4K5
	Cluster 4	7	Regulation of cortical dendrite branching
	Cluster 5	7	Ameloblast differentiation
	Cluster 6	6	Mixed, incl. Adamantinoma, and AJAP1/PANP, C-terminal
	Cluster 7	6	Type I hemidesmosome assembly
	Cluster 8	5	CD74, METAP1, MRPL46, PRDX3, SOD2
	Cluster 9	4	Other semaphorin interactions
	Cluster 10	3	Cardiac muscle cell-cardiac muscle cell adhesion
	Cluster 11	3	Regulation of plasma lipoprotein particle levels
	Cluster 12	3	DNA double-strand break processing
	Cluster 13	3	PNPT1, CLPP, ATP5IF1
	Cluster 14	3	Retrograde trans-synaptic signaling
	Cluster 15	2	Mixed, incl. Mitochondrial fusion, and Mitochondrial dynamics protein MID49/MID51
	Cluster 16	2	Sin3 complex
	Cluster 17	2	Phosphocreatine biosynthesis
	Cluster 18	2	A conserved domain of 46 amino acids, called WHEP-TRS has been shown PUBMED:1756734 to exist in a number of higher eukaryote aminoacyl-transfer RNA synthetases. This domain is present one to six times in the several enzymes.

Supplementary Fig. 3 | STRING network analysis of tear film dysregulation in the post-COVID-19 persistent ocular symptoms (POS) group reveals cluster adaptive immunity protein dysregulation. Markov clustering of 178 dysregulated tear film proteins by STRING network analysis, with color-coded clusters and gene counts within clusters, in descending order. Disconnected nodes are not displayed. Solid lines indicate within-cluster and dashed lines between-cluster interactions. Line colors: pink – experimentally-determined, teal – from curated databases, green – predicted from gene neighborhood, red – predicted from gene fusions, blue – gene co-occurrence, yellow – text mining, black – co-expression, grey – protein homology.

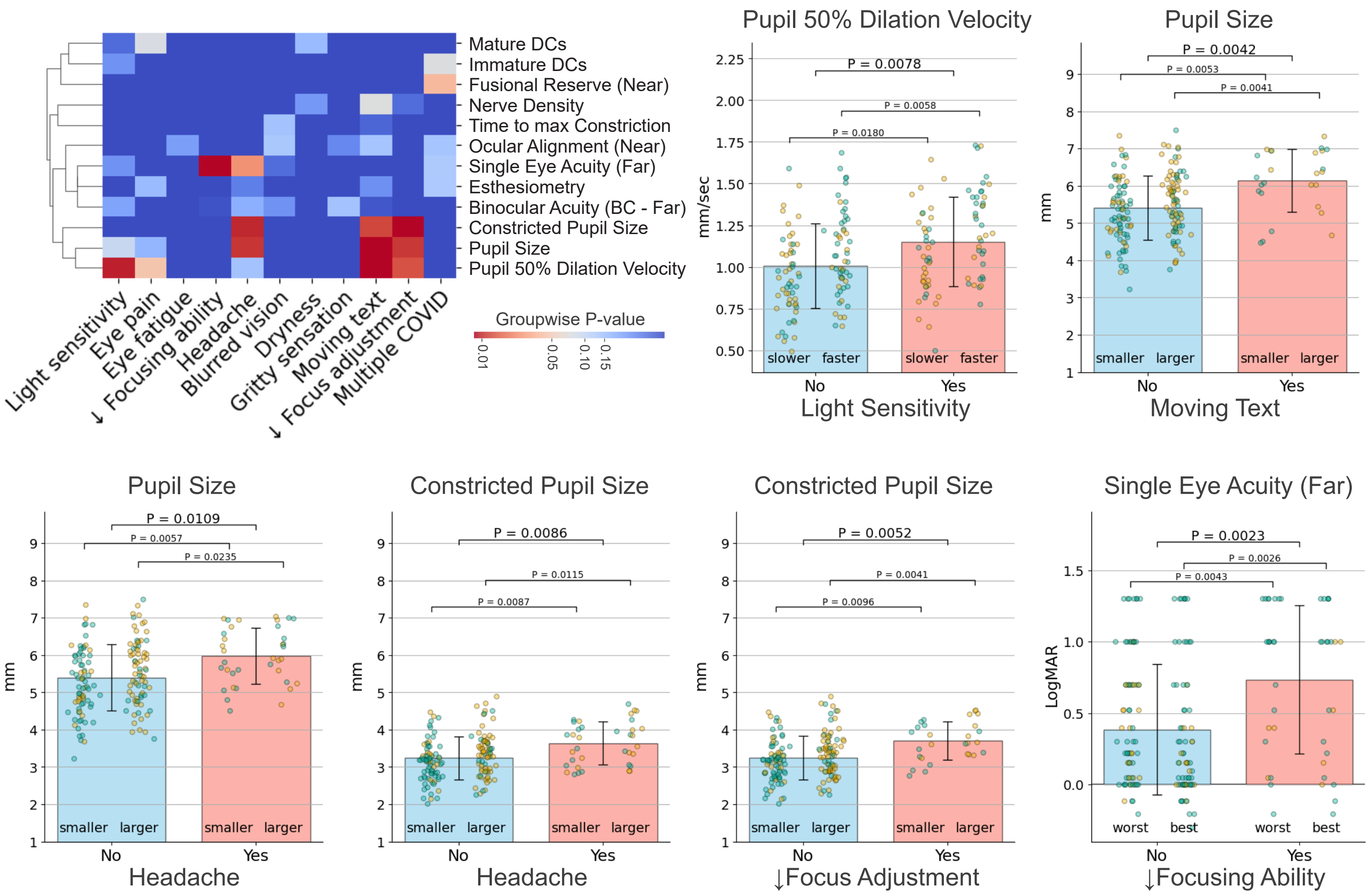

Supplementary Fig. 4 | Pupillary parameters correlate with reported presence of various post-COVID-19 persistent ocular symptoms (POS). Heat map illustrates the significance of groupwise comparisons of the presence of various self-reported POS with ocular parameter values. Charts indicate associations of subpopulations with (Yes) or without (No) various POS with pupil parameters and monocular distance acuity. LogMAR: logarithm of the minimum angle of resolution; DCs: dendritic/T cells; BC: best corrected binocular acuity.

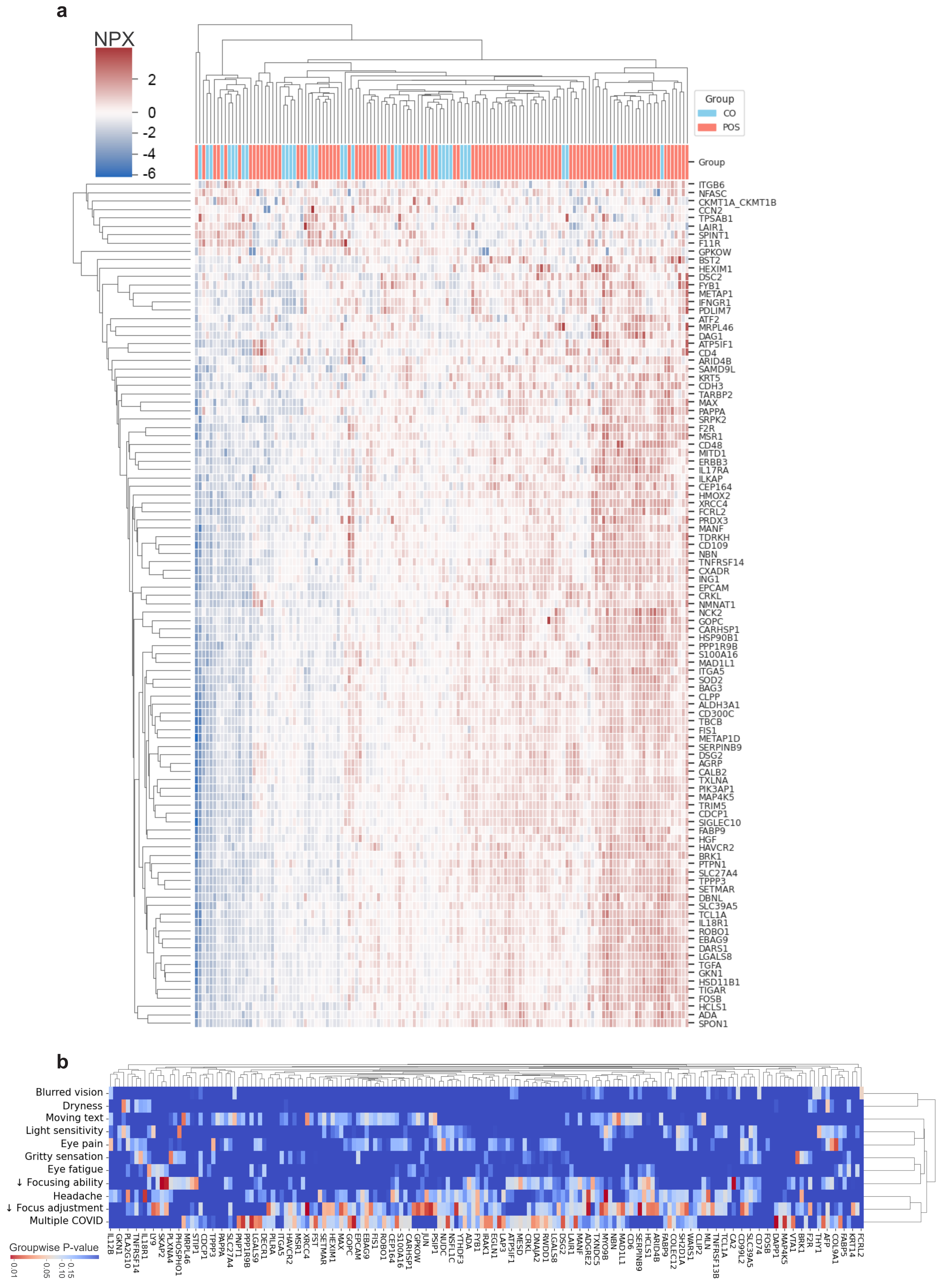
a
NPX
2
0
-2
-4
-6
Group
CO
POS
Group
ITGB6
NFASC
CKMT1A_CKMT1B
CCN2
TPSAB1
LAIR1
SPINT1
F11R
GPKOW
BST2
HEXIM1
DSC2
FYB1
METAP1
IFNGR1
PDLIM7
ATF2
MRPL46
DAG1
ATP5IF1
CD4
ARID4B
SAMD9L
KRT5
CDH3
TARBP2
MAX
PAPPA
SRPK2
F2R
MSR1
CD48
MITD1
ERBB3
IL17RA
ILKAP
CEP164
HMOX2
XRCC4
FCRL2
PRDX3
MANF
TDRKH
CD109
NBN
TNFRSF14
CXADR
ING1
EPCAM
CRKL
NMNAT1
NCK2
GOPC
CARHSP1
HSP90B1
PPP1R9B
S100A16
MAD1L1
ITGA5
SOD2
BAG3
CLPP
ALDH3A1
CD300C
TBCB
FIS1
METAP1D
SERPINB9
DSG2
AGRP
CALB2
TXLNA
PIK3AP1
MAP4K5
TRIM5
CDCP1
SIGLEC10
FABP9
HGF
HAVCR2
BRK1
PTPN1
SLC27A4
TPPP3
SETMAR
DBNL
SLC39A5
TCL1A
IL18R1
ROBO1
EBAG9
DARS1
LGALS8
TGFA
GKN1
HSD11B1
TIGAR
FOSB
HCLS1
ADA
SPON1
b
Blurred vision
Dryness
Moving text
Light sensitivity
Eye pain
Gritty sensation
Eye fatigue
↓ Focusing ability
Headache
↓ Focus adjustment
Multiple COVID
Groupwise P-value
0.01
0.05
0.10
0.15
IL12B
GKN1
PLA2G10
TNFRSF14
IL18R1
LY9
SKAP2
PLXNA4
PHOSPHO1
MRPL46
STIP1
CDCP1
TPPP3
PAPPA
SLC27A4
PNPT1
PPP1R9B
LGALS9
DECR1
PILRA
ITGA5
HAVCR2
MSR1
XRCC4
FST
SETMAR
HEXIM1
MAX
GOPC
EPCAM
EBAG9
FIS1
ROBO1
CEP164
S100A16
CARHSP1
GPKOW
JUN
PSIP1
NUDC
NSFL1C
YTHDF3
ADA
FYB1
IRAK1
EGLN1
LAP3
ATP5IF1
MESD
CRKL
DNAJA2
RWDD1
LGALS8
DSG2
LAIR1
MANF
ADGRE2
TXNDC5
MYO9B
NBN
MAD1L1
CD6
SERPINB9
HCLS1
ARID4B
FABP9
COLEC12
SH2D1A
WARS1
CLIP2
MLN
TNFRSF13B
TCL1A
CA2
CD99L2
SLC39A5
CD74
FOSB
DAPP1
MAP4K5
VTA1
BRK1
F2R
THY1
AFP
COL9A1
FABP5
KRT14
FCRL2

Supplementary Fig. 5 | Unbiased hierarchical clustering of dysregulated tear proteins separates post-COVID-19 persistent ocular symptoms (POS) from control (CO) subjects. (**a**) Cluster dendrograms show hierarchical relationships between dysregulated tear film proteins and between expression patterns for all study participants, with each pixel representing the Olink normalized protein expression level (NPX, heatmap color-coded). Proteins had high expression in POS and low expression in CO. (**b**) Heatmap showing unadjusted significance of symptom presence with expression levels of dysregulated proteins in POS.

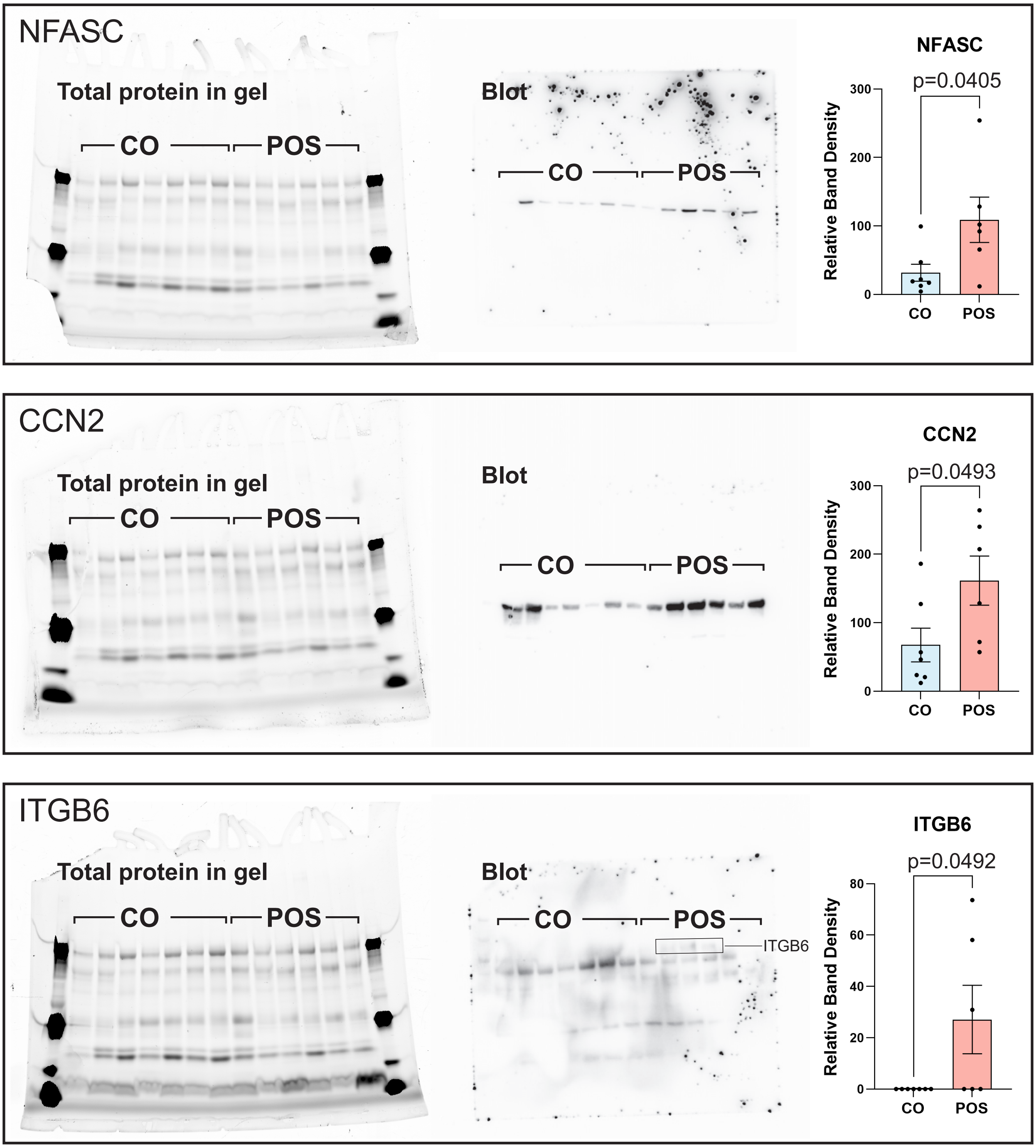

Supplementary Fig. 6 | Limited validation of tear film proteomic data by Western blot. Whole gel and whole blot images of NFASC, CCN2 and ITGB6 blots. Top row: NFASC detection, middle row: CCN2 detection, bottom row: ITGB6 detection. ITGB6 was faintly detectable in 3 post-COVID-19 persistent ocular symptoms (POS) group samples, with multiple unspecific bands at smaller sizes across all lanes. Densitometry was performed, data were normalized versus total protein density, and two-tailed groupwise t-tests were conducted (respective panels to the right). All bar graphs present data as mean values ± Standard Deviation, overlaid with single data points per band (sample). CO: control samples.

Supplementary Table 1 | Participant Demographic data

	Persistent ocular symptoms (POS)	Control (CO)	P-value
Group size (n)	100	32	
Sex<br>F - M, ratio	73F - 27M, F/M=2.7	24F - 8M, F/M=3	$\chi^2(1)$ 0.823
Age (y)<br>mean±SD / median (min-max)	48±14.2 / 49 (5-75)	50±18.4 / 54 (13-75)	t-test 0.568
Time from 1st Covid infection<br>(wks from examination day)<br>mean±SD / median (min-max)	97±39.1 / 106 (15-158)	93±51.3 / 98 (11-180)	t-test 0.605
Time from last Covid infection<br>(weeks from examination day)<br>mean±SD / median (min-max)	73±42.3 / 67 (3-158)	63±47.3 / 57 (9-158)	t-test 0.249
Eye symptom onset delay (weeks)<br>mean±SD / median (min-max)	16±15.8 / 11 (1-57)		
Eye symptom duration at examination (weeks)<br>mean±SD / median (min-max)	87±39.2 / 88 (17-152)		

Supplementary Table 2 | Significant proteomic marker levels and statistics

Assay	UniProt	OlinkID	Panel	Mean CO	Mean POS	Log2 Fold Change	p-value	Adjusted p (Benjamini-Hochberg FDR 0.05)
ITGB6	P18564	OID20493	Inflammation	-0.5423	0.1163	0.6585	3.9E-05	0.0271
NFASC	O94856	OID20634	Inflammation	-0.1049	0.1105	0.2154	7.5E-05	0.0271
CKMT1A_CKMT1B	P12532	OID20721	Inflammation	-0.7759	0.0152	0.7910	0.0001	0.0290
CCN2	P29279	OID20709	Inflammation	-0.2009	0.0389	0.2398	0.0002	0.0350
TPSAB1	Q15661	OID20642	Inflammation	-0.6004	0.8113	1.4117	0.0003	0.0436
SPINT1	O43278	OID21109	Neurology	-0.3940	-0.0255	0.3685	0.0005	0.0558
LAIR1	Q6GTX8	OID20737	Inflammation	-0.8043	0.0061	0.8103	0.0006	0.0592
F11R	Q9Y624	OID21151	Neurology	-0.2666	0.0016	0.2683	0.0009	0.0839
GPKOW	Q92917	OID20890	Neurology	-0.4157	0.1060	0.5217	0.0012	0.0899
BST2	Q10589	OID21029	Neurology	-0.3208	0.2957	0.6165	0.0014	0.0899
HEXIM1	O94992	OID20589	Inflammation	-0.5931	0.0275	0.6206	0.0014	0.0899
DSC2	Q02487	OID21079	Neurology	-0.4815	0.0662	0.5476	0.0015	0.0903
FYB1	O15117	OID21140	Neurology	-0.6684	0.0533	0.7216	0.0017	0.0903
METAP1	P53582	OID20815	Neurology	0.1332	-0.0815	-0.2147	0.0020	0.0903
IFNGR1	P15260	OID20678	Inflammation	-0.2549	0.0398	0.2947	0.0023	0.0903
PDLIM7	Q9NR12	OID20729	Inflammation	-0.6211	0.0386	0.6597	0.0024	0.0903
ATF2	P15336	OID20798	Neurology	-0.3372	0.1278	0.4650	0.0025	0.0903
MRPL46	Q9H2W6	OID20858	Neurology	-0.1290	0.1757	0.3047	0.0027	0.0903
DAG1	Q14118	OID20774	Inflammation	-0.5190	-0.0095	0.5096	0.0028	0.0903
ATP5IF1	Q9UII2	OID20760	Inflammation	-0.7194	-0.1098	0.6096	0.0029	0.0903
CD4	P01730	OID20584	Inflammation	-0.2749	0.0947	0.3696	0.0030	0.0903
ARID4B	Q4LE39	OID20803	Neurology	-0.3827	0.0761	0.4588	0.0032	0.0903
SAMD9L	Q8IVG5	OID20536	Inflammation	-0.4909	0.0288	0.5196	0.0033	0.0903
KRT5	P13647	OID20860	Neurology	-0.3262	0.0217	0.3480	0.0034	0.0903
CDH3	P22223	OID21008	Neurology	-0.3987	0.0509	0.4495	0.0035	0.0903
TARBP2	Q15633	OID20870	Neurology	-0.3387	0.0321	0.3707	0.0035	0.0903
MAX	P61244	OID20877	Neurology	-0.6069	0.0246	0.6315	0.0037	0.0903
PAPPA	Q13219	OID20421	Inflammation	-0.5540	-0.0251	0.5290	0.0038	0.0903
SRPK2	P78362	OID20502	Inflammation	-0.4731	0.0042	0.4773	0.0039	0.0903
F2R	P25116	OID20691	Inflammation	-0.2602	0.0892	0.3494	0.0040	0.0903
MSR1	P21757	OID21063	Neurology	-0.5483	0.0619	0.6102	0.0040	0.0903
CD48	P09326	OID20692	Inflammation	-0.4951	0.0905	0.5856	0.0040	0.0903
MITD1	Q8WV92	OID20959	Neurology	-0.3428	-0.0101	0.3327	0.0042	0.0903

ERBB3	P21860	OID20705	Inflammation	-0.2873	0.0208	0.3081	0.0043	0.0903
IL17RA	Q96F46	OID21089	Neurology	-0.5378	-0.0275	0.5103	0.0046	0.0903
ILKAP	Q9H0C8	OID20855	Neurology	-0.4673	0.0069	0.4742	0.0047	0.0903
CEP164	Q9UPV0	OID20441	Inflammation	-0.3253	0.1042	0.4295	0.0047	0.0903
HMOX2	P30519	OID20887	Neurology	-0.5072	0.0028	0.5099	0.0048	0.0903
XRCC4	Q13426	OID20869	Neurology	-0.2433	0.1014	0.3446	0.0049	0.0903
FCRL2	Q96LA5	OID20639	Inflammation	-0.3776	0.1435	0.5211	0.0050	0.0903
PRDX3	P30048	OID20483	Inflammation	-0.2391	0.1862	0.4253	0.0051	0.0903
MANF	P55145	OID20707	Inflammation	-0.5736	-0.0382	0.5354	0.0052	0.0903
TDRKH	Q9Y2W6	OID20875	Neurology	-0.4452	0.0719	0.5172	0.0054	0.0913
TNFRSF14	Q92956	OID20783	Inflammation	-0.2918	0.0140	0.3058	0.0058	0.0950
CD109	Q6YHK3	OID21108	Neurology	-0.4163	0.0319	0.4482	0.0060	0.0953
NBN	O60934	OID20442	Inflammation	-0.3873	0.2564	0.6437	0.0062	0.0953
CXADR	P78310	OID20561	Inflammation	-0.3325	0.1401	0.4726	0.0063	0.0953
ING1	Q9UK53	OID20853	Neurology	-0.4954	0.0571	0.5525	0.0066	0.0953
EPCAM	P16422	OID20743	Inflammation	-0.4386	-0.0376	0.4010	0.0066	0.0953
ALDH3A1	P30838	OID20512	Inflammation	0.0535	-0.0375	-0.0909	0.0067	0.0953
CRKL	P46109	OID20742	Inflammation	-0.5229	-0.0994	0.4235	0.0068	0.0953
NMNAT1	Q9HAN9	OID20913	Neurology	-0.4296	0.1159	0.5455	0.0068	0.0953
NCK2	O43639	OID20683	Inflammation	-0.4400	0.0043	0.4443	0.0070	0.0958
GOPC	Q9HD26	OID20551	Inflammation	-0.4332	-0.0291	0.4041	0.0076	0.0979
CARHSP1	Q9Y2V2	OID20859	Neurology	-0.4110	-0.0432	0.3677	0.0076	0.0979
HSP90B1	P14625	OID20829	Neurology	0.5357	-0.2120	-0.7477	0.0080	0.0979
PPP1R9B	Q96SB3	OID20643	Inflammation	-0.4510	-0.0392	0.4118	0.0082	0.0979
AGRP	O00253	OID20658	Inflammation	0.5084	-0.0307	-0.5391	0.0082	0.0979
S100A16	Q96FQ6	OID20878	Neurology	-0.4702	-0.0712	0.3990	0.0084	0.0979
MAD1L1	Q9Y6D9	OID20904	Neurology	-0.3454	0.0766	0.4220	0.0085	0.0979
DSG2	Q14126	OID21113	Neurology	-0.4701	-0.0819	0.3881	0.0085	0.0979
ITGA5	P08648	OID21139	Neurology	-0.6902	-0.0901	0.6002	0.0086	0.0979
SOD2	P04179	OID21114	Neurology	-0.4801	-0.0794	0.4007	0.0086	0.0979
BAG3	O95817	OID20972	Neurology	-0.3769	-0.0060	0.3709	0.0087	0.0979
CLPP	Q16740	OID20910	Neurology	-0.3980	0.1120	0.5099	0.0089	0.0979
CD300C	Q08708	OID21010	Neurology	-0.3331	0.1502	0.4833	0.0090	0.0979
TBCB	Q99426	OID20993	Neurology	-0.5052	-0.0433	0.4619	0.0090	0.0979
FIS1	Q9Y3D6	OID20722	Inflammation	-0.4892	-0.1022	0.3871	0.0097	0.1033
METAP1D	Q6UB28	OID20519	Inflammation	-0.4196	0.1249	0.5445	0.0100	0.1044
SERPINB9	P50453	OID20932	Neurology	-0.3197	0.0261	0.3458	0.0101	0.1044

CALB2	P22676	OID20801	Neurology	0.1338	-0.2713	-0.4051	0.0105	0.1076
TXLNA	P40222	OID20952	Neurology	-0.4556	-0.0362	0.4194	0.0111	0.1108
PIK3AP1	Q6ZUJ8	OID20587	Inflammation	-0.3563	0.1324	0.4887	0.0112	0.1108
FABP9	Q0Z7S8	OID20501	Inflammation	0.4381	-0.0812	-0.5193	0.0115	0.1115
MAP4K5	Q9Y4K4	OID20899	Neurology	-0.4540	-0.0337	0.4203	0.0115	0.1115
TRIM5	Q9C035	OID20526	Inflammation	-0.3027	0.0481	0.3508	0.0120	0.1145
SLC27A4	Q6P1M0	OID20814	Neurology	-0.4358	0.0425	0.4782	0.0125	0.1175
CDCP1	Q9H5V8	OID20940	Neurology	-0.2253	0.0533	0.2786	0.0126	0.1175
SIGLEC10	Q96LC7	OID20537	Inflammation	-0.1477	0.2659	0.4136	0.0129	0.1177
HGF	P14210	OID20656	Inflammation	-0.3545	0.0211	0.3757	0.0131	0.1177
HAVCR2	Q8TDQ0	OID21137	Neurology	-0.6603	-0.1023	0.5580	0.0132	0.1177
SLC39A5	Q6ZMH5	OID20539	Inflammation	0.2082	-0.0365	-0.2446	0.0136	0.1203
BRK1	Q8WUW1	OID20883	Neurology	-0.2516	0.0363	0.2879	0.0138	0.1203
PTPN1	P18031	OID20908	Neurology	-0.3837	0.0493	0.4330	0.0140	0.1207
TPPP3	Q9BW30	OID20842	Neurology	-0.4987	-0.0964	0.4023	0.0148	0.1265
SETMAR	Q53H47	OID20948	Neurology	-0.4257	0.0519	0.4776	0.0151	0.1271
TCL1A	P56279	OID20987	Neurology	-0.3992	0.5078	0.9070	0.0154	0.1278
DBNL	Q9UJU6	OID20681	Inflammation	-0.3960	-0.0795	0.3165	0.0155	0.1278
IL18R1	Q13478	OID20652	Inflammation	-0.1920	0.0665	0.2585	0.0161	0.1309
ROBO1	Q9Y6N7	OID20734	Inflammation	-0.5623	-0.0488	0.5135	0.0171	0.1367
EBAG9	O00559	OID20918	Neurology	-0.4526	-0.0118	0.4408	0.0172	0.1367
GKN1	Q9NS71	OID20885	Neurology	0.0888	-0.3985	-0.4873	0.0176	0.1380
DARS1	P14868	OID20903	Neurology	-0.3805	-0.0589	0.3215	0.0177	0.1380
LGALS8	O00214	OID21041	Neurology	-0.3793	-0.0784	0.3009	0.0180	0.1382
TGFA	P01135	OID20600	Inflammation	-0.2202	0.0644	0.2846	0.0181	0.1382
HSD11B1	P28845	OID20575	Inflammation	0.2107	-0.1118	-0.3225	0.0185	0.1389
TIGAR	Q9NQ88	OID20894	Neurology	-0.3014	-0.0451	0.2563	0.0186	0.1389
FOSB	P53539	OID20791	Neurology	-0.3875	0.1449	0.5324	0.0190	0.1402
HCLS1	P14317	OID20648	Inflammation	-0.4020	0.0179	0.4199	0.0194	0.1413
ADA	P00813	OID20645	Inflammation	-0.4322	-0.0027	0.4295	0.0196	0.1413
SPON1	Q9HCB6	OID20759	Inflammation	-0.6492	-0.1294	0.5198	0.0197	0.1413
FST	P19883	OID20770	Inflammation	-0.4755	-0.1063	0.3692	0.0202	0.1413
KLRB1	Q12918	OID20629	Inflammation	-0.2767	0.1663	0.4430	0.0204	0.1413
FKBP5	Q13451	OID20937	Neurology	-0.6006	-0.0197	0.5809	0.0204	0.1413
KRT14	P02533	OID20800	Neurology	-0.3042	0.0680	0.3722	0.0205	0.1413
WARS1	P23381	OID21084	Neurology	0.0682	-0.1190	-0.1872	0.0207	0.1413
CD99L2	Q8TCZ2	OID21122	Neurology	-0.2657	-0.0001	0.2656	0.0220	0.1492

CD40	P25942	OID20724	Inflammation	-0.2366	0.0165	0.2532	0.0225	0.1510
PLXNA4	Q9HCM2	OID20597	Inflammation	-0.9706	-0.2915	0.6791	0.0240	0.1573
RWDD1	Q9H446	OID21015	Neurology	-0.3353	-0.0910	0.2443	0.0241	0.1573
IL18	Q14116	OID20694	Inflammation	-0.3126	-0.0208	0.2919	0.0242	0.1573
PREB	Q9HCU5	OID20439	Inflammation	-0.0732	0.0464	0.1196	0.0244	0.1573
YTHDF3	Q7Z739	OID20478	Inflammation	-0.2926	0.0149	0.3075	0.0245	0.1573
ADGRE2	Q9UHX3	OID20755	Inflammation	-0.4356	-0.0801	0.3555	0.0253	0.1607
DNAJA2	O60884	OID20627	Inflammation	-0.3543	-0.0903	0.2641	0.0258	0.1607
NOS1	P29475	OID20863	Neurology	-0.7788	0.0880	0.8668	0.0258	0.1607
CD6	P30203	OID20649	Inflammation	-0.4691	0.0991	0.5683	0.0259	0.1607
IL12B	P29460	OID20666	Inflammation	-0.2618	0.1736	0.4354	0.0263	0.1607
LAYN	Q6UX15	OID20970	Neurology	-0.1574	0.1583	0.3156	0.0264	0.1607
EGLN1	Q9GZT9	OID20572	Inflammation	-0.4853	-0.0900	0.3952	0.0269	0.1627
IRAK1	P51617	OID20485	Inflammation	-0.3809	-0.0646	0.3164	0.0273	0.1633
LAP3	P28838	OID20436	Inflammation	-0.4347	-0.0857	0.3490	0.0277	0.1649
SH2D1A	O60880	OID20514	Inflammation	-0.4013	0.1275	0.5289	0.0280	0.1650
COL9A1	P20849	OID20550	Inflammation	0.1823	-0.0151	-0.1973	0.0302	0.1740
LBR	Q14739	OID21034	Neurology	-0.3839	0.1668	0.5507	0.0303	0.1740
IL16	Q14005	OID20633	Inflammation	-0.4591	0.0850	0.5442	0.0304	0.1740
JUN	P05412	OID20424	Inflammation	-0.2152	0.0928	0.3080	0.0307	0.1740
CD58	P19256	OID20716	Inflammation	-0.2407	0.0524	0.2931	0.0310	0.1740
TBC1D17	Q9HA65	OID20844	Neurology	-0.2699	-0.0247	0.2452	0.0313	0.1740
FRZB	Q92765	OID20968	Neurology	-0.2578	0.1005	0.3582	0.0314	0.1740
MGMT	P16455	OID20588	Inflammation	-0.5204	-0.1240	0.3965	0.0314	0.1740
NUDC	Q9Y266	OID20623	Inflammation	-0.2827	-0.0683	0.2144	0.0318	0.1742
VSIG4	Q9Y279	OID21144	Neurology	-0.5593	-0.0489	0.5104	0.0322	0.1742
MESD	Q14696	OID21099	Neurology	-0.4872	-0.1186	0.3686	0.0324	0.1742
MZB1	Q8WU39	OID20732	Inflammation	-0.1834	0.3582	0.5416	0.0327	0.1742
CDON	Q4KMG0	OID20754	Inflammation	-0.3416	-0.0132	0.3284	0.0328	0.1742
MASP1	P48740	OID20954	Neurology	0.0656	-0.0736	-0.1392	0.0331	0.1742
VTA1	Q9NP79	OID21016	Neurology	-0.2935	-0.0763	0.2173	0.0336	0.1742
TREML2	Q5T2D2	OID21120	Neurology	-0.4830	-0.0198	0.4631	0.0336	0.1742
SERPINB6	P35237	OID21023	Neurology	-0.2261	-0.0585	0.1676	0.0338	0.1742
TNFRSF13B	O14836	OID20702	Inflammation	-0.1943	0.2258	0.4202	0.0341	0.1742
STIP1	P31948	OID21077	Neurology	-0.2715	-0.0640	0.2075	0.0341	0.1742
RASA1	P20936	OID20826	Neurology	0.0498	-0.0478	-0.0976	0.0350	0.1759
CD74	P04233	OID21040	Neurology	-0.1596	0.0172	0.1768	0.0350	0.1759

SKAP2	O75563	OID20761	Inflammation	-0.3859	0.2170	0.6029	0.0352	0.1759
PPCDC	Q96CD2	OID21027	Neurology	-0.3436	-0.0786	0.2650	0.0355	0.1760
HARS1	P12081	OID21086	Neurology	-0.2869	-0.0828	0.2041	0.0361	0.1767
MYO9B	Q13459	OID20451	Inflammation	-0.3814	-0.0612	0.3202	0.0362	0.1767
ANGPTL2	Q9UKU9	OID20726	Inflammation	-0.2913	0.0579	0.3492	0.0363	0.1767
COLEC12	Q5KU26	OID20738	Inflammation	-0.4927	-0.0988	0.3939	0.0374	0.1778
IL33	O95760	OID20428	Inflammation	-0.4276	0.1866	0.6142	0.0376	0.1778
LY9	Q9HBG7	OID20670	Inflammation	-0.1491	0.0477	0.1968	0.0378	0.1778
PILRA	Q9UKJ1	OID21129	Neurology	-0.3744	-0.0071	0.3674	0.0378	0.1778
DAPP1	Q9UN19	OID20524	Inflammation	-0.4108	-0.0270	0.3838	0.0380	0.1778
CCS	O14618	OID20973	Neurology	-0.2596	0.0058	0.2654	0.0382	0.1778
GP6	Q9HCN6	OID21091	Neurology	-0.4916	-0.0552	0.4364	0.0383	0.1778
PSIP1	O75475	OID20534	Inflammation	-0.5147	0.0179	0.5325	0.0386	0.1783
NSFL1C	Q9UNZ2	OID21022	Neurology	-0.2210	-0.0199	0.2011	0.0416	0.1909
PHOSPHO1	Q8TCT1	OID20922	Neurology	0.1813	0.0037	-0.1776	0.0419	0.1909
TXNDC5	Q8NBS9	OID21012	Neurology	-0.3378	-0.0518	0.2860	0.0423	0.1912
MLN	P12872	OID20541	Inflammation	0.1106	-0.2777	-0.3883	0.0426	0.1912
AFP	P02771	OID20978	Neurology	-0.4975	-0.0744	0.4232	0.0429	0.1912
TST	Q16762	OID20868	Neurology	-0.2025	0.0945	0.2969	0.0430	0.1912
BMP4	P12644	OID20994	Neurology	0.2528	-0.1573	-0.4101	0.0432	0.1912
PLA2G10	O15496	OID21002	Neurology	0.1104	-0.2016	-0.3120	0.0438	0.1923
SEMA4D	Q92854	OID21020	Neurology	-0.3600	-0.0172	0.3428	0.0449	0.1952
CA2	P00918	OID21149	Neurology	-0.4662	0.0664	0.5326	0.0451	0.1952
THY1	P04216	OID21050	Neurology	-0.3044	0.0143	0.3186	0.0452	0.1952
FABP5	Q01469	OID21043	Neurology	0.1157	-0.0342	-0.1499	0.0456	0.1954
LGALS9	O00182	OID20781	Inflammation	-0.3725	-0.0803	0.2922	0.0463	0.1964
SERPINB8	P50452	OID20630	Inflammation	-0.2093	-0.0293	0.1800	0.0463	0.1964
DECR1	Q16698	OID20579	Inflammation	-0.3539	0.0578	0.4117	0.0466	0.1966
CLIP2	Q9UDT6	OID20559	Inflammation	-0.3679	0.0038	0.3717	0.0470	0.1970
VCAN	P13611	OID21026	Neurology	0.2259	-0.0788	-0.3047	0.0474	0.1974
PLA2G7	Q13093	OID21105	Neurology	-0.3688	-0.0308	0.3380	0.0478	0.1979
CXCL1	P09341	OID20762	Inflammation	-0.3339	-0.0520	0.2819	0.0481	0.1981
CASP10	Q92851	OID20893	Neurology	-0.2224	-0.0424	0.1800	0.0495	0.2026
PNPT1	Q8TCS8	OID20445	Inflammation	-0.1277	0.1418	0.2695	0.0498	0.2028

Supplementary Table 3 | Diagnostic Model Parameters

5 clinical parameter model - AUC: 0.77

Feature	Coefficient	Mean	Std	(Example) Data input	Excel pseudo-formula
Esthesiometry (mm)	-0.92897	58.230769	4.7010457	60	=1 / (1 + EXP(-(1.611640
Pupil 50% Dilation Velocity (mm/sec)	0.4026055	1.0574658	0.2579318	1.55	- 0.928970 * (Esthesiometry - 58.2308) / 4.701
Time to Minimum Pupil Size (sec)	0.3190547	1.3572627	0.1562464	1.495	+ 0.402605 * (Pupil_50%_Dilation_Velocity - 1.0575) / 0.2579
Subbasal Nerve Density (mm/mm^2)	-0.71171	18.863871	3.4871211	13.6907	+ 0.319055 * (Time_to_Minimum_Pupil_Size - 1.3573) / 0.1562
Mature DC/Ts (cells/mm^2)	1.2589607	10.545058	10.28051	8.3565	- 0.711710 * (Subbasal_Nerve_Density - 18.8639) / 3.4871
Intercept	1.6116399				+ 1.258961 * (Mature_DC/Ts - 10.5451) / 10.2805)))

Copy-paste-ready excel formula for the above table layout, with top left cell ("Feature") placed on position A1:

=1 / (1 + EXP(-(B7 + B2 * (E2 - C2) / D2 + B3 * (E3 - C3) / D3 + B4 * (E4 - C4) / D4 + B5 * (E5 - C5) / D5 + B6 * (E6 - C6) / D6)))

Thresholds: Youden's J: 0.8726, Closest to (0,1): 0.7200, TPR > 90%: 0.5265, TPR > 95%: 0.4076, FPR < 5%: 0.8726, FPR < 10%: 0.8529

10 parameter model - AUC: 0.91

Feature	Coefficient	Mean	Std	(Example) Data input	Excel pseudo-formula
Esthesiometry (mm)	-0.934733	58.230769	4.7010457	60	=1 / (1 + EXP(-(2.378509
Pupil 50% Dilation Velocity (mm/sec)	0.9458225	1.0574658	0.2579318	1.55	- 0.934733 * (Esthesiometry - 58.2308) / 4.701
Subbasal Nerve Density (mm/mm^2)	-1.014273	18.863871	3.4871211	13.6907	+ 0.945823 * (Pupil_50%_Dilation_Velocity - 1.0575) / 0.2579
Mature DC/Ts (cells/mm^2)	1.4961043	10.545058	10.28051	8.3565	- 1.014273 * (Subbasal_Nerve_Density - 18.8639) / 3.4871
CCN2	0.8835167	-0.024577	0.4035152	0.9566	+ 1.496104 * (Mature_DC/Ts - 10.5451) / 10.2805
FCRL2	0.9022597	0.0055787	0.9611626	-1.7308	+ 0.883517 * (CCN2 + 0.0246) / 0.4035
TRIM5	0.9947982	-0.044738	0.7203408	0.4846	+ 0.902260 * (FCRL2 - 0.0056) / 0.9612
BMP4	-0.538616	-0.048765	1.0422154	0.3592	+ 0.994798 * (TRIM5 + 0.0447) / 0.7203
VCAN	-1.145595	0.0018463	0.78907	-0.3075	- 0.538616 * (BMP4 + 0.0488) / 1.0422
PLA2G10	-0.950268	-0.118996	0.8137243	0.678	- 1.145595 * (VCAN - 0.0018) / 0.7891
Intercept	2.378509				- 0.950268 * (PLA2G10 + 0.1190) / 0.8137)))

Copy-paste-ready excel formula for the above table layout, with top left cell ("Feature") placed on position A1:

=1 / (1 + EXP(-(B12 + B2 * (E2 - C2) / D2 + B3 * (E3 - C3) / D3 + B4 * (E4 - C4) / D4 + B5 * (E5 -C5) / D5 + B6 * (E6 - C6) / D6 + B7 * (E7 - C7) / D7 + B8 * (E8 - C8) / D8 + B9 * (E9 - C9) / D9 + B10 * (E10 - C10) / D10 + B11 * (E11 - C11) / D11)))

Thresholds: Youden's J: 0.8074, Closest to (0,1): 0.7200, TPR > 90%: 0.5282, TPR > 95%: 0.3921, FPR < 5%: 0.9793, FPR < 10%: 0.9172

Supplementary Table 4 | Modified Catquest-9SF questionnaire and scoring

**Questions**	**Options**	**Score**
**Global assessment items**		
A. Do you find that your sight at the present in some way causes you difficulty in everyday life?	1. Yes, very great difficulty<br>2. Yes, great difficulty<br>3. Yes, some difficulty<br>4. No, no difficulty<br>5. Can't decide	4<br>3<br>2<br>1
B. Are you satisfied or dissatisfied with your present vision?	1. Very dissatisfied<br>2. Fairly dissatisfied<br>3. Fairly satisfied<br>4. Very satisfied<br>5. Can't decide	4<br>3<br>2<br>1
**Difficulty items:**<br>*Do you have difficulty with the following activities because of your vision? If so, to what extent?*		
C1 - Reading text in the newspaper	1. Yes, very great difficulty<br>2. Yes, great difficulty<br>3. Yes, some difficulty<br>4. No, no difficulty<br>5. Can't decide	4<br>3<br>2<br>1
C2 - Recognizing faces of people you meet		
C3 - Seeing the prices of goods when shopping		
C4 - Seeing to walk on uneven surfaces, e.g. cobblestones		
C5 - Seeing to do needlework and handicrafts		
C6 - Reading from screens		
C7 - Seeing to carry out a preferred hobby		

Supplementary Table 5 | Group sizes, statistical model selection per clinical parameter and significance values

Parameter	N CO	N POS	F-test p-value	Equal Variance	Age Model Comparison p	Preferred Model	Preferred Model p	MDES (Cohen d)	Simulated Power (d=0.6)
Corneal Thickness	32	100	0.960188	TRUE	0.914599	No-age model	0.726929	0.57325	0.817
Corneal Thickness min	32	100	0.775422	TRUE	0.792558	No-age model	0.813936	0.57325	0.832
Corneal Thickness max	32	100	0.882889	TRUE	0.970848	No-age model	0.653816	0.57325	0.837
Endothelial Cell Density	32	98	0.484397	TRUE	2.30×10^{-9}	Age-model	0.681099	0.57473	0.83
Endothelial Cell Density min	32	98	0.174402	TRUE	3.98×10^{-9}	Age-model	0.524758	0.57473	0.828
Endothelial Cell Density max	32	98	0.909026	TRUE	3.16×10^{-8}	Age-model	0.871602	0.57473	0.834
NIKBUT	32	100	0.433734	TRUE	0.203284	No-age model	0.52726	0.57325	0.812
NIKBUT min	32	100	0.294894	TRUE	0.087203	No-age model	0.309209	0.57325	0.812
NIKBUT max	32	100	0.935568	TRUE	0.422012	No-age model	0.793989	0.57325	0.826
Redness	32	99	0.734568	TRUE	0.000001	Age-model	0.482357	0.57398	0.841
Redness min	32	99	0.971785	TRUE	4.12×10^{-10}	Age-model	0.571045	0.57398	0.83
Redness max	32	99	0.694193	TRUE	0.000567	Age-model	0.514559	0.57398	0.816
Near Point of Accommodation	32	99	0.219594	TRUE	1.57×10^{-34}	Age-model	0.25312	0.57398	0.828
Schirmer	32	98	0.314676	TRUE	0.007281	Age-model	0.203022	0.57473	0.836
Schirmer min	32	98	0.232386	TRUE	0.007718	Age-model	0.138015	0.57473	0.822
Schirmer max	32	98	0.753556	TRUE	0.009681	Age-model	0.305293	0.57473	0.815
Tear Meniscus	32	100	0.481389	TRUE	0.013421	Age-model	0.224391	0.57325	0.819
Tear Meniscus min	32	100	0.873713	TRUE	0.049108	Age-model	0.121883	0.57325	0.816
Tear Meniscus max	32	100	0.377134	TRUE	0.009808	Age-model	0.380668	0.57325	0.827
Esthesiometry	31	99	0.001618	FALSE	0.442478	No-age model	0.002227	0.58097	0.814
Esthesiometry min	31	99	0.000741	FALSE	0.288577	No-age model	0.002913	0.58097	0.813
Esthesiometry max	31	99	0.005732	FALSE	0.9465	No-age model	0.01301	0.58097	0.832
Binocular Acuity - Near - Best correction	32	100	0.000618	FALSE	0.867451	No-age model	0.00472	0.57325	0.831
Binocular Acuity - Near - No correction	32	100	0.150467	TRUE	1.34×10^{-8}	Age-model	0.069322	0.57325	0.847
Binocular Acuity - Far - Best correction	32	100	0.178161	TRUE	0.514234	No-age model	0.210992	0.57325	0.842
Binocular Acuity - Far - No correction	32	100	0.058205	TRUE	0.008648	Age-model	0.064084	0.57325	0.825
Single Eye Acuity - Far - Best correction	32	100	0.220229	TRUE	0.325452	No-age model	0.230598	0.57325	0.825

Single Eye Acuity - Far - Best correction min	32	100	0.136905	TRUE	0.760666	No-age model	0.40548	0.57325	0.827
Single Eye Acuity - Far - Best correction max	32	100	0.374256	TRUE	0.148518	No-age model	0.168668	0.57325	0.82
Single Eye Acuity - Far - No correction	32	100	0.069842	TRUE	0.0004	Age-model	0.022464	0.57325	0.814
Single Eye Acuity - Far - No correction min	32	100	0.009811	FALSE	0.000542	Age-model	0.012769	0.57325	0.812
Single Eye Acuity - Far - No correction max	32	100	0.123277	TRUE	0.000126	Age-model	0.01582	0.57325	0.83
Ocular Alignment (Far)	32	100	0.264959	TRUE	0.298085	No-age model	0.584242	0.57325	0.832
Ocular Alignment (Near)	32	100	0.000009	FALSE	0.918117	No-age model	0.000774	0.57325	0.828
Stereoacuity (TNO)	32	100	0.055361	TRUE	0.056987	No-age model	0.089691	0.57325	0.803
Fusional Reserve (Far)	32	99	0.051552	TRUE	0.479849	No-age model	0.832718	0.57398	0.814
Fusional Reserve (Near)	32	99	0.100497	TRUE	0.659599	No-age model	0.014418	0.57398	0.83
Refractive Error - sphere (auto)	32	99	0.062435	TRUE	0.00051	Age-model	0.084216	0.57398	0.827
Refractive Error - sphere min (auto)	32	99	0.048852	FALSE	0.000227	Age-model	0.052886	0.57398	0.811
Refractive Error - sphere max (auto)	32	99	0.055402	TRUE	0.000223	Age-model	0.078207	0.57398	0.822
Refractive Error - sphere (subjective)	32	100	0.06099	TRUE	0.007556	Age-model	0.080809	0.57325	0.847
Refractive Error - sphere (subjective) min	32	100	0.058142	TRUE	0.029617	Age-model	0.136785	0.57325	0.815
Refractive Error - sphere (subjective) max	32	100	0.052033	TRUE	0.002459	Age-model	0.054591	0.57325	0.849
Refractive Error - cylinder (auto)	32	99	0.949526	TRUE	0.083472	No-age model	0.698031	0.57398	0.809
Refractive Error - cylinder min (auto)	32	99	0.728616	TRUE	0.221427	No-age model	0.92642	0.57398	0.824
Refractive Error - cylinder max (auto)	32	99	0.866241	TRUE	0.046957	Age-model	0.506793	0.57398	0.827
Refractive Error - cylinder (subjective)	32	100	0.556654	TRUE	0.018177	Age-model	0.764635	0.57325	0.81
Refractive Error - cylinder (subjective) min	32	100	0.593828	TRUE	0.114469	No-age model	0.953912	0.57325	0.82
Refractive Error - cylinder (subjective) max	32	100	0.62603	TRUE	0.003456	Age-model	0.671408	0.57325	0.851
Pupil Size	31	97	0.002385	FALSE	5.27×10^{-17}	Age-model	0.025887	0.58247	0.82
Pupil Size min	31	97	0.004618	FALSE	1.39×10^{-16}	Age-model	0.022932	0.58247	0.822
Pupil Size max	31	97	0.00239	FALSE	6.20×10^{-17}	Age-model	0.031479	0.58247	0.835
Constricted Pupil Size	31	97	0.165122	TRUE	2.21×10^{-13}	Age-model	0.009543	0.58247	0.802
Constricted Pupil Size min	31	97	0.167058	TRUE	7.52×10^{-13}	Age-model	0.014385	0.58247	0.799
Constricted Pupil Size max	31	97	0.204939	TRUE	5.08×10^{-13}	Age-model	0.008978	0.58247	0.817
Pupil Size Change on Stimulus	31	97	0.00029	FALSE	0.139599	No-age model	0.699034	0.58247	0.816
Pupil Size Change on Stimulus min	31	97	0.0046	FALSE	0.199045	No-age model	0.885268	0.58247	0.812

Pupil Size Change on Stimulus max	31	97	0.000209	FALSE	0.11728	No-age model	0.523666	0.58247	0.817
PLR Latency	31	97	0.235405	TRUE	0.002401	Age-model	0.549563	0.58247	0.794
PLR Latency min	31	97	0.040169	FALSE	0.003573	Age-model	0.206676	0.58247	0.812
PLR Latency max	31	97	0.833699	TRUE	0.00954	Age-model	0.885312	0.58247	0.842
Pupil 50% Dilation Velocity	31	97	0.743673	TRUE	9.25×10^{-9}	Age-model	0.016751	0.58247	0.825
Pupil 50% Dilation Velocity min	31	97	0.884486	TRUE	1.60×10^{-7}	Age-model	0.040895	0.58247	0.833
Pupil 50% Dilation Velocity max	31	97	0.371774	TRUE	4.30×10^{-9}	Age-model	0.010281	0.58247	0.794
Time to Minimum Pupil Size	31	97	0.000999	FALSE	0.002045	Age-model	0.012957	0.58247	0.794
Time to Minimum Pupil Size min	31	97	0.006342	FALSE	0.004241	Age-model	0.045828	0.58247	0.822
Time to Minimum Pupil Size max	31	97	0.000748	FALSE	0.001662	Age-model	0.003251	0.58247	0.81
Time to Pupil 50% Recovery	31	97	0.000276	FALSE	0.012139	Age-model	0.278672	0.58247	0.83
Time to Pupil 50% Recovery min	31	97	0.000304	FALSE	0.005821	Age-model	0.248188	0.58247	0.784
Time to Pupil 50% Recovery max	31	97	0.001261	FALSE	0.0306	Age-model	0.321512	0.58247	0.825
Nerve Density	30	97	0.093733	TRUE	0.718326	No-age model	0.000112	0.58982	0.817
Nerve Density min	30	97	0.020817	FALSE	0.861644	No-age model	0.000964	0.58982	0.802
Nerve Density max	30	97	0.228034	TRUE	0.610034	No-age model	0.000157	0.58982	0.836
Immature DCs	30	99	0.515755	TRUE	0.145195	No-age model	0.562497	0.58834	0.807
Immature DCs min	30	99	0.928744	TRUE	0.069441	No-age model	0.901461	0.58834	0.799
Immature DCs max	30	99	0.239042	TRUE	0.256072	No-age model	0.315461	0.58834	0.8
Mature DCs	30	99	0.000494	FALSE	0.480075	No-age model	2.49×10^{-9}	0.58834	0.804
Mature DCs min	30	99	0.001376	FALSE	0.436804	No-age model	6.64×10^{-8}	0.58834	0.798
Mature DCs max	30	99	0.000311	FALSE	0.529144	No-age model	9.39×10^{-10}	0.58834	0.816
All DCs	30	99	0.440411	TRUE	0.122317	No-age model	0.272483	0.58834	0.813
All DCs min	30	99	0.796083	TRUE	0.056231	No-age model	0.645021	0.58834	0.819
All DCs max	30	99	0.209396	TRUE	0.229127	No-age model	0.147198	0.58834	0.792
