# Supplementary material for: Long-term ocular symptoms following COVID-19 linked to immune dysregulation, dysautonomia and peripheral neuropathy
Source: Nat Commun. 2026 Jul 8;17:5624. doi: 10.1038/s41467-026-74858-4 (PMC13346420; doi:10.1038/s41467-026-74858-4)
Supplement: Supplementary file 3 — Reporting Summary [file 41467_2026_74858_MOESM3_ESM.pdf]

nature portfolio

Corresponding author(s): Neil Lagali

Last updated by author(s): May 21, 2026

Reporting Summary

Nature Portfolio wishes to improve the reproducibility of the work that we publish. This form provides structure for consistency and transparency in reporting. For further information on Nature Portfolio policies, see our Editorial Policies and the Editorial Policy Checklist.

Statistics

For all statistical analyses, confirm that the following items are present in the figure legend, table legend, main text, or Methods section.

n/a	Confirmed	
☐	☒	The exact sample size (*n*) for each experimental group/condition, given as a discrete number and unit of measurement
☐	☒	A statement on whether measurements were taken from distinct samples or whether the same sample was measured repeatedly
☐	☒	The statistical test(s) used AND whether they are one- or two-sided<br>*Only common tests should be described solely by name; describe more complex techniques in the Methods section.*
☐	☒	A description of all covariates tested
☐	☒	A description of any assumptions or corrections, such as tests of normality and adjustment for multiple comparisons
☐	☒	A full description of the statistical parameters including central tendency (e.g. means) or other basic estimates (e.g. regression coefficient) AND variation (e.g. standard deviation) or associated estimates of uncertainty (e.g. confidence intervals)
☒	☐	For null hypothesis testing, the test statistic (e.g. *F*, *t*, *r*) with confidence intervals, effect sizes, degrees of freedom and *P* value noted<br>*Give P values as exact values whenever suitable.*
☒	☐	For Bayesian analysis, information on the choice of priors and Markov chain Monte Carlo settings
☐	☒	For hierarchical and complex designs, identification of the appropriate level for tests and full reporting of outcomes
☐	☒	Estimates of effect sizes (e.g. Cohen's *d*, Pearson's *r*), indicating how they were calculated

Our web collection on statistics for biologists contains articles on many of the points above.

Software and code

Policy information about availability of computer code

Data collection	For collection of clinical data by in vivo confocal microscopy, the built-in Heidelberg Eye Explorer v.1.9.13.0 was used. For specular microscopy, Topcon IMAGEnet i-base software v.3.12.1 was used with SP Cell Count Module v.1.19.0. For optical coherence tomography, the built-in software Optovue iVue v.3.2 was used. For the Oculus Keratograph 5M, the built-in Patient Management Software v.6.08r13 was used. For dynamic pupillometry, the built-in software v.28-0.26-0.15-0004 was used.
Data analysis	For Rasch analysis of Catquest 9SF responses, the WINSTEPS v.4.0.1 (https://www.winsteps.com/winsteps.htm) and jMetrik v.4.1.1 software (open source https://itemanalysis.com/jmetrik-download/) was used.<br>For proteomic data analyses, the free online STRING (https://string-db.org/) and Wikipathways (https://www.wikipathways.org/) platforms were used. Statistical analyses and models were generated from the open source Python libraries pandas (v 2.3.3), statsmodels (v 0.14.6) and scipy (v 1.16.3).

For manuscripts utilizing custom algorithms or software that are central to the research but not yet described in published literature, software must be made available to editors and reviewers. We strongly encourage code deposition in a community repository (e.g. GitHub). See the Nature Portfolio guidelines for submitting code & software for further information.

Data

Policy information about availability of data

All manuscripts must include a data availability statement. This statement should provide the following information, where applicable:

- Accession codes, unique identifiers, or web links for publicly available datasets
- A description of any restrictions on data availability
- For clinical datasets or third party data, please ensure that the statement adheres to our policy

Source data for all figure panels are provided with this paper as per-panel .csv files in a zipped Source Data container file. Individual, anonymized source data and IVCM images have been deposited in the Figshare database under accession code 10.6084/m9.figshare.30245398. The Swedish Health Authority national statistics on COVID-19 were sourced from and are also available at https://www.folkhalsomyndigheten.se/faktablad/fall-covid-19/. The Swedish–translated version of the Catquest-9SF survey and its layout design as presented to the participants is available upon request. All processed data generated in this study are provided in the Supplementary Information and the Source Data file.

Research involving human participants, their data, or biological material

Policy information about studies with human participants or human data. See also policy information about sex, gender (identity/presentation), and sexual orientation and race, ethnicity and racism.

Reporting on sex and gender	Sex has been reported in the manuscript and in the raw data. Sex and gender were not considered in the study design or as criteria for inclusion in the observational study. Sex and/or gender of participants was determined based on self reporting. Overall numbers and disaggregated numbers for sex and gender are provided in the paper and in the source data files.
Reporting on race, ethnicity, or other socially relevant groupings	Not reported.
Population characteristics	Demographic data are provided in Supplementary Table 1
Recruitment	Subjects from the general population with specific ocular health concerns after COVID-19 contacted the study investigators for possible inclusion in the study following broad Swedish media reporting (newspaper, radio, television and internet) of the proposed study. Subjects were subsequently screened for confirmed COVID-19 diagnosis based on positive qPCR test result, or during the period of Feb-May 2020 when tests were not widely available in Sweden, a strong suspicion of COVID-19 infection based on acute symptoms (fever, loss of smell or taste, fatigue, headache, sore throat, nasal congestion) and presence of risk factors (such as travel to region with reported outbreak or symptomatic infection within the household). Medical records and screening questions were used to confirm the absence of prior ocular diagnoses, ocular surgeries, or chronic diseases with possible ocular manifestation including diabetes and other systemic autoimmune or neurodegenerative diseases. Inclusion criteria were: aged 4 years and older (for compliance with testing procedures), had recovered from mild COVID-19 without hospitalization, and debut of visual or ocular symptoms persisting after infection and with duration of at least 12 weeks at the time of examination. Exclusion criteria were history of prior ocular surgery or eye disease, severe ocular comorbidities not related to the anterior eye (such as age-related macular degeneration, retinal detachment, optic nerve pathology, etc.), eye deformities, and history of daily contact lens wear (past short-term or intermittent contact use lens use in the past, for example during sporting activities, was however, allowed). 32 age-matched volunteers with prior SARS-CoV-2 infection but no prior or current ocular symptoms or systemic comorbidities were recruited and served as controls.
Ethics oversight	All participants provided voluntary written signed informed consent to participate in the study. The study was initiated following approval by the Swedish Ethical Review Authority (approval nos. 2022-00365-01 and 2022-04607-02) and adhered to the tenets of the Declaration of Helsinki, October 2013.

Note that full information on the approval of the study protocol must also be provided in the manuscript.

Field-specific reporting

Please select the one below that is the best fit for your research. If you are not sure, read the appropriate sections before making your selection.

☒ Life sciences ☐ Behavioural & social sciences ☐ Ecological, evolutionary & environmental sciences

For a reference copy of the document with all sections, see nature.com/documents/nr-reporting-summary-flat.pdf

Life sciences study design

All studies must disclose on these points even when the disclosure is negative.

Sample size	Sample sizes were fixed based on the number of subjects that could be recruited and examined for the study during an 18 month inclusion period from April 2022 to Sept 2023. Sample sizes were validated by post-hoc power calculations for each study parameter individually.
Data exclusions	No data were excluded from the analyses.
Replication	Multiple discrete clinical measurements and images were taken, in both eyes, and results were averaged or included data from both eyes applying the appropriate statistical correction. For in vivo confocal microscopy images, image review, selection and quantitative assessment steps were repeated by two trained experienced observers masked to each other. Full manual analyses were performed on separately

selected image datasets for corneal subbsal nerves and dendritic cells to improve robustness of the data analysis. Proteomics data was analyzed with technical replicates. We also replicated the Olink proteomic data for a selection of top dysregulated proteins and individuals in both groups (minimum individuals 6 per group) using the antibody-based Western blot.

Randomization

Participants were included in consecutive order of inclusion, with inclusion randomized with respect to CO or EXP groups. Proteomics analysis was completed by randomized distribution of samples across sample plates.

Blinding

Investigators were blinded to the participant group during data extraction, compilation and subsequent analysis prior to groupwise analyses; however, data collection was not blinded as investigators were required to collect data regarding infection status, medications and verify patient-provided information during the clinical study visit. Furthermore, the ease of examinations and patient compliance/tolerance to examinations was dependent on symptom burden in a manner that was not amenable to blinding. Many patients within the POS group were highly sensitive to light and wore sunglasses indoors, and/or experienced constant eye pain that could not be hidden from view of the investigators during clinical examinations.

Reporting for specific materials, systems and methods

We require information from authors about some types of materials, experimental systems and methods used in many studies. Here, indicate whether each material, system or method listed is relevant to your study. If you are not sure if a list item applies to your research, read the appropriate section before selecting a response.

Materials & experimental systems

n/a	Involved in the study	
☐	☒	Antibodies
☒	☐	Eukaryotic cell lines
☒	☐	Palaeontology and archaeology
☒	☐	Animals and other organisms
☐	☒	Clinical data
☒	☐	Dual use research of concern
☒	☐	Plants

Methods

n/a	Involved in the study	
☒	☐	ChIP-seq
☒	☐	Flow cytometry
☒	☐	MRI-based neuroimaging

Antibodies

Antibodies used

Mouse monoclonal anti-CTGF antibody | AB01/1E5 IgG2a from Biorad. Catalog# VMA00685. Lot# 64584198, Neurofascin (NFASC) Mouse Monoclonal Antibody [Clone ID: OTI11A7] from Origene. Catalog # TA815269S. Lot# F001, ITGB6 Mouse Monoclonal Antibody [Clone ID: OTI1D2] from Origene. Catalog # TA507354S. Lot# F001, Goat anti-Mouse IgG (H+L) Poly-HRP Secondary Antibody from Invitrogen via ThermoFisher Scientific. Product ref 32230 Lot# ZB387677.

Validation

CCN2 antibody validated for Western Blot by Bio-rad:
https://www.bio-rad-antibodies.com/monoclonal/human-ctgf-antibody-ab01-1e5-vma00685.html?f=purified&WT.mc_id=250116044919
Recommended dilution: max 1:1000, dilution used: 1:500

NFASC antibody validated for Western Blot by Origene: https://www.origene.com/catalog/antibodies/primary-antibodies/ta815269s-neurofascin-nfasc-mouse-monoclonal-antibody-clone-id-oti11a7
Recommended dilution for WB: 1:2000, dilution used: 1:500

ITGB6 antiboldy validated for Western Blot by Origene: https://www.origene.com/catalog/antibodies/primary-antibodies/ta507354s-itgb6-mouse-monoclonal-antibody-clone-id-oti1d2
Recommended dilution for WB: 1:1000, dilution used: 1:500

Secondary anti-mouse IgG validated for Western blot by us (used routinely) and also by the company and in publications listed on the website:
https://www.thermofisher.com/antibody/product/Goat-anti-Mouse-IgG-H-L-Poly-HRP-Secondary-Antibody-Polyclonal/32230
Recommended dilution for WB: 1:5000 – 1:20000, dilution used: 1:2000

Clinical data

Policy information about clinical studies
All manuscripts should comply with the ICMJE guidelines for publication of clinical research and a completed CONSORT checklist must be included with all submissions.

Clinical trial registration

This was not an interventional clinical trial, it was an observational study.

Study protocol

The full trial protocol is written in Swedish and is available from the authors upon request.

Data collection

Clinical examinations were conducted at the Eye Clinic, Linköping University Hospital at the Department of Biomedical and Clinical

Sciences, Linköping University, with all study visits conducted between April 2022 and September 2023 and all clinical examinations performed by the same investigators.

Participants were recruited from eye clinics, optometrists, post-COVID clinics, and from the general public after broad media reporting and advertising in the local newspaper. Presumptive participants were screened by telephone and medical records prior to study inclusion. Participants were included based on self-reported symptoms prior to objective testing, as there was a lack of diagnostic criteria available. This could constitute a self-selection bias, with the most affected individuals seeking medical care and being most prone to contact the investigators. Furthermore, a majority of females were included in the study, which may be a further self-selection bias, as being more prone to seeking help or answers. This may have resulted in an over-representation of females or more severe eye-related symptoms following COVID-19, and we cannot exclude the possibility of existence of a group of individuals with milder eye symptoms following COVID-19 that who did not seek medical attention, and who were not included in the study.

Outcomes

Outcome measures were based on a predefined set of clinical parameters outlined in the study protocol, without a priori identification of primary or secondary parameters. All outcomes were reported equally. Covariate cohort characteristics such as age, sex, and COVID-19 infection data have been documented and described in Supplementary Table 1.

Plants

Seed stocks

not applicable

Novel plant genotypes

not applicable

Authentication

not applicable
