# Supplementary material for: Long-term ocular symptoms following COVID-19 linked to immune dysregulation, dysautonomia and peripheral neuropathy
Source: Nat Commun. 2026 Jul 8;17:5624. doi: 10.1038/s41467-026-74858-4 (PMC13346420; doi:10.1038/s41467-026-74858-4)
Supplement: Supplementary file 2 — Peer Review file [file 41467_2026_74858_MOESM2_ESM.pdf]

nature portfolio

Peer Review File

Long-term ocular symptoms following COVID-19 linked to immune dysregulation, dysautonomia and peripheral neuropathy

Corresponding Author: Professor Neil Lagali

This file contains all reviewer reports in order by version, followed by all author rebuttals in order by version.

Version 0:

Reviewer comments:

Reviewer #1

(Remarks to the Author)
The authors examined in a prospective cross-sectional study persistent ocular symptoms (POS) emerging after mild COVID-19 and documented ocular symptoms persisting from 3 months up to 3 years post-infection. It is shown that POS lead to significant vision disability and are linked to clinical findings only seen with specialized ophthalmic testing. POS are characterized by near vision disturbances, strabismus, weakened autonomic blink and pupillary constriction reflexes, corneal neurodegeneration and chronic activation of ocular surface dendritic/T cells. Proteomics analysis detected a tear film signature consistent with severe COVID-19, chronic dysregulation of CD4+ T cell regulatory activity and upregulation of proteins ITGB6, NFASC, CTGF, TPSAB1 and CKMT1A-CKMT1B. Pupil dysfunction correlated with JUN, and dendritic/T cell dysregulation correlated with ANGPTL2, SKAP2 and DAPP1. The authors constructed individual-level diagnostic models based on clinical examinations with or without protein biomarkers. These models predict POS with 77-91% accuracy and implicate chronic T cell-mediated neuroinflammation in the pathogenesis of POS, a debilitating syndrome arising after COVID-19 recovery. The data are important especially that ocular sequelae of COVID-19 have been often downplayed by physicians. The paper actually provides a reason for this oversight due to the need of special examination going beyond routine eye exams. In this respect, the study is really important and very significant for the ophthalmology and infectious diseases field. This reviewer has some concerns below that the authors are invited to address.
1. In the abstract, it is unclear what the control individuals were, and what their vaccination status was. Additionally, the protein of interest should be listed as up- or down-regulated, not “correlated with” (under pupil dysfunction; the same in the main text). The first sentence should be changed as there are still many serious cases of COVD-19, and generally speaking the disease is not mild. The authors should specify why they call it mild: because of the latest attenuated status of the virus or because the ocular symptoms are generally mild.
2. As changes of the posterior eye are also known, they deserve some mention as well.
3. The title is too broad as the paper deals mostly with the ocular surface.
4. It is unclear why the study populations are heavily shifted toward female participants.
5. Correlation of dilated and constricted pupil size with transcription factor JUN seems odd and needs better explanation. In this respect, the state of the lacrimal glands including possible damage by the disease may be of interest.
6. The proteomics data need some verification by western blot as the method is based on peptide detection.
7. Another limitation is the role of immune cells in long COVID sequelae in the eye. The authors assume changes in T cells and dendritic cells based on tear markers and morphology, but it remains to be formally established whether the actual cells expressing specific markers of their respective lineages are accumulating or being activated in the cornea and/or lacrimal gland.
8. Due to a relatively low number of patients acknowledged by the authors, an additional look at the data by a biostatistician may be warranted for.
9. Certain box plots have two parameters in each box, which makes it difficult to assess changes in disease. Please consider separating two entities, e.g., on Fig. 2 and supplementals.

Reviewer #2

(Remarks to the Author)

Moustardas and colleagues describe a group of PC patients in comparison to normals with persisting ocular sequelae.
The text is clearly written and methods elaborate. Two points of discussion:
1. Age of PC patients ranged from five years to 75 years. Could You comment on the number of children with young age included in the study. If the normal group was age matched, the youngest participant would be still 13 years old, more than double of the five year old from the PC group. Furthermore how could a child could manage the Catquest-9SF without reading abilities or deeper understanding of the test content. Were both parents involved?
2. RCM showed reduced density in the subepithelial plexus. As we know, that the ROI is rather small, how did the authors could achieve, that the same region of the cornea was imaged and measured in all subjects. Did the authors successfully investigate the youngest?
3. What was the reason not to investigate vessel density of the retina with OCTA if light sensitivity was a main complain and could be triggered by the retina as in visual snow syndrome?
4. If strabism was an issue, why not use Harms'screen instead of rather rough Bagolini glasses?
5. Was reported strabism related to a special group of muscles and cranial nerves?
6. Could the authors perform a test to measure light sensitivity in PC?
Thank You

Reviewer #3

(Remarks to the Author)
The authors have a very technical and detailed product from their extensive work, which I commend them for. However, the overall design of the study is methodologically flawed. The primary purpose of the study is to compare persons with POS to persons without POS following COVID infection. Therefore, essentially participants are assigned to one of these two groups based on self-report of their POS, and we would obviously expect participants with POS to have differences in their measures of ocular health since they are self-reporting ocular conditions! When reading this paper, the question that kept coming up for me - Would we see these same differences in a comparison of persons with POS versus not in the general population, regardless of COVID infection? If so, then why is COVID at the center of this study? There are other possibilities for a control group, of which I would recommend two: persons with POS without a COVID diagnosis, or persons without POS and without a COVID diagnosis. Including both of these control groups would help us better understand if it is COVID that is in fact causing differences in the measures of interest, or if it is simply the POS. As the paper is currently designed, all we know is some of these measures differ for persons reporting POS compared to persons not reporting POS, which doesn't seem all that interesting or important.

In addition to the above issue, there is way too much data included in the report and it is very easy to get lost. Good research has a more targeted research question, and it feels overwhelming to see all of this information together. Some items I was confused about:
1. Why are person measures between the two groups compared. They already self-selected into groups as having vision issues, so of course they differ in their self-reported vision.
2. Gender differences in symptoms are graphed, but I couldn't find mention of it in the text. Are these differences significant? If not, they should not be included. If they are, the text should mention this.
3. Then there are a bunch of figures that I can't make much sense of. Are these all important? Could they be in a table in order to digest easier?
4. Given the methodologic issues mentioned above, ROC curves are not appropriate.

Version 1:

Reviewer comments:

Reviewer #1

(Remarks to the Author)
The authors provided exhaustive replies to the critiques. This reviewer has no further concerns.

Reviewer #4

(Remarks to the Author)
none

Open Access This Peer Review File is licensed under a Creative Commons Attribution 4.0 International License, which permits use, sharing, adaptation, distribution and reproduction in any medium or format, as long as you give appropriate credit to the original author(s) and the source, provide a link to the Creative Commons license, and indicate if changes were made.
In cases where reviewers are anonymous, credit should be given to 'Anonymous Referee' and the source.
The images or other third party material in this Peer Review File are included in the article's Creative Commons license, unless indicated otherwise in a credit line to the material. If material is not included in the article's Creative Commons license and your intended use is not permitted by statutory regulation or exceeds the permitted use, you will need to obtain permission directly from the copyright holder.
To view a copy of this license, visit https://creativecommons.org/licenses/by/4.0/

REVIEWER COMMENTS

Reviewer #1 (Remarks to the Author):

The authors examined in a prospective cross-sectional study persistent ocular symptoms (POS) emerging after mild COVID-19 and documented ocular symptoms persisting from 3 months up to 3 years post-infection. It is shown that POS lead to significant vision disability and are linked to clinical findings only seen with specialized ophthalmic testing. POS are characterized by near vision disturbances, strabismus, weakened autonomic blink and pupillary constriction reflexes, corneal neurodegeneration and chronic activation of ocular surface dendritic/T cells. Proteomics analysis detected a tear film signature consistent with severe COVID-19, chronic dysregulation of CD4+ T cell regulatory activity and upregulation of proteins ITGB6, NFASC, CTGF, TPSAB1 and CKMT1A-CKMT1B. Pupil dysfunction correlated with JUN, and dendritic/T cell dysregulation correlated with ANGPTL2, SKAP2 and DAPP1. The authors constructed individual-level diagnostic models based on clinical examinations with or without protein biomarkers. These models predict POS with 77-91% accuracy and implicate chronic T cell-mediated neuroinflammation in the pathogenesis of POS, a debilitating syndrome arising after COVID-19 recovery. The data are important especially that ocular sequelae of COVID-19 have been often downplayed by physicians. The paper actually provides a reason for this oversight due to the need of special examination going beyond routine eye exams. In this respect, the study is really important and very significant for the ophthalmology and infectious diseases field. This reviewer has some concerns below that the authors are invited to address.

1. In the abstract, it is unclear what the control individuals were, and what their vaccination status was. Additionally, the protein of interest should be listed as up- or down-regulated, not “correlated with” (under pupil dysfunction; the same in the main text). The first sentence should be changed as there are still many serious cases of COVD-19, and generally speaking the disease is not mild. The authors should specify why they call it mild: because of the latest attenuated status of the virus or because the ocular symptoms are generally mild.

Thank you for the comment. The control individuals have now been explicitly stated in the abstract.

Vaccination status was not something we included in the study protocol (as we stated in the limitations section of the discussion); however, the vaccination rate in Sweden is very high and many of the study subjects in both groups voluntarily reported that they had been vaccinated once or more. Studying vaccination effects was not the aim of this study and would require larger numbers of participants to draw meaningful conclusions given the heterogeneity of viral variants and type and frequency of vaccinations received.

In the abstract we list the top upregulated proteins in POS relative to controls, while the correlation data is different, as it corresponds to proteins highly correlated with specific biological/clinical measurements across ALL (POS + CO) subjects. Nevertheless, we checked the data and can confirm that even these proteins were upregulated in POS relative to the control group. As such, we have revised the wording in the abstract to indicate this.

We apologize for the confusion caused by our ‘mild’ terminology. By ‘mild’ COVID-19, we meant cases not requiring hospitalization. To avoid this confusion, we have

removed the term ‘mild’ from the title and adjusted the wording in the abstract and main text to define the POS group less ambiguously. We agree with the reviewer that generally speaking, the disease is not mild; however, we wanted to emphasize that the majority of cases do not require hospitalization.

2. As changes of the posterior eye are also known, they deserve some mention as well.

We thank the reviewer for this comment and have now updated the first paragraph in the main text to mention posterior segment changes and included two references (5 and 6), one of which is a systematic review and meta-analysis.

3. The title is too broad as the paper deals mostly with the ocular surface.

We agree that the study deals partly with the ocular surface, but we also report important changes in the pupillary response and strabismus (extraocular muscles) as well as symptoms comprising the entire visual pathway to the brain. To specify ‘anterior eye’ or ‘ocular surface’ would be too narrow in our opinion. We thus propose to leave ‘ocular symptoms’ in the title and rely on the abstract to convey the type and extent of ocular involvement.

4. It is unclear why the study populations are heavily shifted toward female participants.

Not only in our population, but in other cohorts, it is well established that female sex is a robust and validated independent risk factor for developing long COVID or post-acute sequelae. For example, in the large American RECOVER cohort with 12,276 participants, females had a significantly higher risk of developing long COVID compared with males after adjusting for confounders, see:

Shah DP, Thaweethai T, Karlson EW, et al. Sex Differences in Long COVID. *JAMA Netw Open.* 2025;8(1):e2455430. doi:10.1001/jamanetworkopen.2024.55430

See also the following viewpoint article for further discussion:

Silva J, Iwasaki A. Sex differences in postacute infection syndromes. Sci Transl Med. 2024 Nov 13;16(773):eado2102. doi: 10.1126/scitranslmed.ado2102.

Our reference 3 (Davis et al.) also noted in their international long COVID cohort of 3763 subjects that 79% were women.

To make this point clearer for readers, we have now stated in the introduction that women are known to be overrepresented in long-COVID and post-acute sequelae, referencing both studies above (Shah et al. and Davis et al.).

5. Correlation of dilated and constricted pupil size with transcription factor JUN seems odd and needs better explanation. In this respect, the state of the lacrimal glands including possible damage by the disease may be of interest.

In the discussion we stated that JUN is a major transcription factor activated as part of the peripheral nerve injury and demyelination responses in sensorimotor neuropathies. This suggests that pupillary response impairment could be a result of peripheral nerve injury and subsequent demyelination from COVID-19, given the

strong correlation of higher JUN levels with abnormally large dilated and constricted pupil sizes. It is well known that JUN is a key injury-response factor strongly activated in Schwann cells during demyelination and after peripheral nerve injury, responsible for the transition of Schwann cells to a repair phenotype. We have now modified the statement in the discussion to clarify this and have added two supporting references.

We agree with the comment that the lacrimal glands could be of interest and as an indirect measure of this we had investigated tear fluid production; however, as shown in Figure S1 we did not detect any differences in this parameter (Schirmer test) between the groups. Rather than a direct effect on the lacrimal glands themselves where the tears are produced, the results in the tears likely mirror the systemic changes in the POS group, similar to the other severe-COVID related factors detected in the tears.

Unfortunately, we did not know *a priori* what the findings would be, and we did not include lacrimal gland biopsy in the study protocol, which is an invasive procedure and would also need to be performed in control subjects. However, this could be the focus of a future study.

6. The proteomics data need some verification by western blot as the method is based on peptide detection.

As a general comment, we would like to state that Olink's proximity extension assay (PEA) proteomic platform that we have utilized for protein marker discovery in this study is a validated, highly specific and reproducible technology, with dual recognition antibody binding that minimizes cross-reactivity and supports robust biomarker quantification without the need for orthogonal validation. This performance has been demonstrated across large-scale studies and multiple independent evaluations of the platform's precision and reliability (Wik L, et al.. Mol Cell Proteomics. 2021;20:100168, Assarsson E, et al. PLoS One. 2014 Apr 22;9(4):e95192).

However, we agree with the reviewer that data validation by independent methods only strengthens the confidence of the reported results. To address this, we faced three limitations, and below we describe how we tried to overcome them. First, the tear material analyzed via the Olink technology had been exhausted for the analyses presented in this study. To mitigate this, we utilized our stored samples from each patient's contralateral eye, since for the Olink analysis we had analyzed tears from one randomly chosen eye. Thus, we cannot expect a direct correspondence of results, but should, however, have similar findings if the targets are indeed relevant to POS. Secondly, Western blot is limited to a handful of samples per blot, and 132 samples could not be realistically run and directly compared in a statistically meaningful way. We have thus utilized a 15-well gel that would fit a sufficient amount of total protein load per sample (9µg based on eluted tear protein concentration) and chose 7 CO samples and 6 POS samples to run, plus the ladder. To choose representative samples given the smaller subset, we utilized our 10-parameter POS predictive model and randomly chose CO subjects below the Youden's J threshold, and POS samples above the Youden's J threshold. Lastly, it is unfeasible to detect via Western blot the number of targets that are detectable via Olink, both logistically (number of antibodies required) and based on material availability. We have chosen to use three antibodies against the targets that were top findings in this study and report

our validation results as a new Extended Data Figure S6. We have verified a significant upregulation of NFASC and CCN2, consistent with the Olink data. ITGB6 was much weaker and barely detectable in several samples from both groups; however, using the same densitometry method for quantification, we noted several data points were zero in both groups but a statistical analysis was nevertheless still possible. We present the blot images and quantitative analyses in the Extended Data Figure 6. We believe that the results validate the robustness and general expression pattern of the Olink data. The methods / antibodies used are described in the revised methods section.

7. Another limitation is the role of immune cells in long COVID sequelae in the eye. The authors assume changes in T cells and dendritic cells based on tear markers and morphology, but it remains to be formally established whether the actual cells expressing specific markers of their respective lineages are accumulating or being activated in the cornea and/or lacrimal gland.

We agree with the reviewer that the evidence we obtained non-invasively is limited and does not directly detect marker expression in these cells. However, dendritic/T cell activation in the corneal epithelium imaged by IVCM is an established method used in clinical assessment, and the significantly upregulated T cell factors in the tear fluid, which is in physical contact with the corneal epithelium, is noteworthy. Nevertheless, we appreciate the reviewer's correct assessment and have therefore expanded the limitations paragraph (second last paragraph in the main text) to indicate that direct surface expression of markers on cells remains to be established, as the tear film markers we detected could be mirroring the systemic or lacrimal gland changes and not those directly in the cornea.

8. Due to a relatively low number of patients acknowledged by the authors, an additional look at the data by a biostatistician may be warranted for.

We thank the reviewer for the comment. We have indeed consulted with a biostatistician to determine the appropriate statistical methods to be used given the nature of our data. As a result, we employed a robust method of determining for each parameter the appropriate statistical test and linear regression model to be used. Additionally, prior to performing group comparisons, we compared the fit of two models, one with age as a confounding factor and one without. For each parameter, the appropriate test was applied, and we report both our methodology on determining the appropriate model, and the results for each parameter in the Supplementary Table 5.

Based on the reviewer's comment, and to have a better assessment of the study's power based on actual data, we performed post-hoc parameter-wise power analyses. For each clinical parameter, we computed the minimum detectable effect size (MDES, expressed as Cohen's d) and conducted simulation-based power analyses using the observed sample sizes and variability. Simulated power was estimated for moderate ($d = 0.5$) and moderately large ($d = 0.6$) effect sizes. These results are reported in the revised Supplementary Table 5. The study was well powered to detect moderately large effects, with 90.2% of parameters achieving ≥80% power at $d = 0.6$. Together,

these analyses indicate that the study design was sufficient to detect moderate-to-large group differences, whereas smaller effects may have remained undetected due to limited power.

Accordingly, we have now added the following text to the Methods section under 'Data analysis – Statistics' to indicate the power analysis performed:

Power analyses were performed post-hoc for each study parameter using observed sample sizes and variability. For each, we report the minimum detectable effect size (MDES, Cohen's d) and simulated power for d = 0.6 (Extended Data Table 5).

In the limitations paragraph, 2nd to last paragraph of the main text, we now include the following:

Our post-hoc power testing indicated at an effect size d = 0.6, 90.2% of parameters achieved ≥ 80% power and all parameters achieved ≥ 78.2% power, indicating the study was sufficiently powered to detect moderate-to-large effects, whereas smaller effects would require larger populations to detect.

Finally, we acknowledge assistance from our biostatistician (Mats Fredrikson) in the revised Acknowledgments section.

9. Certain box plots have two parameters in each box, which makes it difficult to assess changes in disease. Please consider separating two entities, e.g., on Fig. 2 and supplementals.

We agree with the reviewer that the inclusion of multiple comparisons within each panel was confusing and difficult to read. We have thus updated all the relevant figures to include panels where only one parameter is compared between CO and POS subjects, and one p-value is reported. Results from more detailed statistical tests between worse eye and best eye for each parameter have been retained in the Extended Data Table 5, for completeness. Overall, all parameters that were significantly different in any or both of worst/best eye comparisons were also significant overall between CO and POS, so no change in the reported results was required.

Reviewer #2 (Remarks to the Author):

Moustardas and colleagues describe a group of PC patients in comparison to normals with persisting ocular sequelae.
The text is clearly written and methods elaborate. Two points of discussion:

1. Age of PC patients ranged from five years to 75 years. Could You comment on the number of children with young age included in the study. If the normal group was age matched, the youngest participant would be still 13 years old, more than double of the five year old from the PC group. Furthermore how could a child could manage the Catquest-9SF without reading abilities or deeper understanding of the test content. Were both parents involved?

We thank the reviewer for the comment and the insight on age-matching our cohorts. Indeed, the youngest participant in the POS group was 5 years old, and was assisted by legal guardians throughout the process; however, the Catquest-9SF was not completed for this participant. This young age was only one case, as the next oldest participant was 13 years old. Importantly, mean age and age distribution did not differ significantly between our two cohorts, making them comparable, even if for certain parameters, an age confounding factor model was used. Following the reviewer's comment, we have updated the manuscript to explicitly state that there were 6 participants with POS under the age of 18 at the time of examination. We have also revised the participant recruitment part of the methods to clarify that for those under the age of 18, signed consent from all legal guardians was required for inclusion.

2. RCM showed reduced density in the subepithelial plexus. As we know, that the ROI is rather small, how did the authors could achieve, that the same region of the cornea was imaged and measured in all subjects. Did the authors successfully investigate the youngest?

We thank the reviewer for mentioning this point. To maintain reproducibility, the imaging was performed by a single operator for all subjects, having 20+ years of experience with this technique, ensuring that a large enough central zone was imaged in all subjects for subsequent image selection. Unfortunately, however, it was not possible to investigate the youngest participant with this method. Typically, only children over the age of 10 are sufficiently compliant for the examination to be performed.

We also agree that it is important to standardize the region for the image analysis. The images of the subbasal plexus obtained in this study were used to select a set of images from the central cornea, using the anatomic 'whorl' feature as a landmark. The imaging was performed such that a series of images could be scanned to determine the position of images relative to the whorl. Specifically, five distinct images superior to the whorl with a predominantly vertical orientation and without overlap were chosen. By this means, we could be confident that approximately the same anatomic area of the central cornea was analyzed. This selection procedure was repeated independently by two observers to yield two different selected datasets which were then analyzed separately and later averaged. The same anatomic region was identified in a similar manner for inflammatory cell image selection and quantification. We have now updated the final paragraph in the 'Clinical Ophthalmic Examinations' section of the Methods to better describe the standardized images that were selected for the analysis.

3. What was the reason not to investigate vessel density of the retina with OCTA if light sensitivity was a main complain and could be triggered by the retina as in visual snow syndrome?

When developing the protocol for the clinical examinations, we did not have a priori knowledge of the range and type of symptoms we would uncover. As we only had anecdotal information of symptoms from a small number of patients prior to initiating the study, we decided to address where the knowledge gap was greatest, and thus focused on possible neural dysregulation in the anterior eye and ocular muscles, instead of retinal microvascular changes which had already been reported and reviewed in conjunction with COVID-19. We additionally had to limit the number of clinical examinations to conduct in participants, as our protocol required participants

to undergo 1.5-hours of ocular examinations which proved to be challenging for POS subjects. We noted only one case of visual snow syndrome in the POS group.

4. If strabism was an issue, why not use Harms'screen instead of rather rough Bagolini glasses?

We thank the reviewer for this insight. Unfortunately, we did not know *a priori* what the findings would uncover, i.e., we did not know we would find strabismus in the POS group. In our protocol we opted for the Bagolini test as a first screening test that evaluated binocular function and fusion under normal, daily viewing conditions. The results, however, motivate the use of Harms test in a future study, for deeper investigation into the specific angular deviations and isolating which extraocular muscles are most affected.

5. Was reported strabism related to a special group of muscles and cranial nerves?

As noted above, we did not use the Harms test for determination of possible affected muscles and thereby the regulating cranial nerves. The esophoria we detected in POS indicates an imbalance of neuromuscular control but whether this is a result of overaccommodation, lateral rectus pathology, or fusion problems cannot be determined by the tests we conducted. Further imaging (eg. MRI) could confirm possible cranial neuropathies.

6. Could the authors perform a test to measure light sensitivity in PC?

Unfortunately, there is no universal accepted quantitative test for measurement of light sensitivity/photophobia. Instead, questionnaires can be used like the Utah Photophobia Symptom Impact Scale, and pupillary light reaction can be performed. Although we did not know *a priori* that light sensitivity would be a major symptom of POS, fortunately we included quantitative dynamic pupillometry as part of the examination protocol. The results confirm weakened pupillary regulation in POS; however, we did not perform static light exposure testing while measuring blink frequency and reflex tearing. After uncovering photophobia as a major symptom in POS, we can motivate further studies using these additional approaches.

Thank You

Reviewer #3 (Remarks to the Author):

The authors have a very technical and detailed product from their extensive work, which I commend them for. However, the overall design of the study is methodologically flawed. The primary purpose of the study is to compare persons with POS to persons without POS following COVID infection. Therefore, essentially participants are assigned to one of these two groups based on self-report of their POS, and we would obviously expect participants with POS to have differences in their measures of ocular health since they are self-reporting ocular conditions!

We thank the reviewer for this observation. Our aim was to investigate patients presenting with POS, which is self-reported and symptom-based, with the goal of

finding objective and test-based differences and potential biomarkers of POS. This can be likened to long COVID, which is also based on self-reported symptoms, and has later been confirmed by clinical and molecular testing.

At the outset of our study there was no means to detect the presence of POS other than self-reported symptoms (similar to long COVID). A methodical approach was taken, first describing the pattern of symptoms experienced by this group, and next exploring the severity of the symptoms and resulting impact on quality of life, which was the purpose of the Catquest questionnaire. As pointed out, the finding that the POS group differs in ocular health measures from those without POS is not surprising, but it is a necessary step to establish the nature of such differences and their severity and impact on quality of life.

The Catquest 9SF is a validated and well-established tool to evaluate self-reported ocular health and is widely used for quantifying self-reported measures (using Rasch analysis) and was originally developed for cataract surgery. Obviously, cataract patients will differ from controls, but it is nonetheless important to establish their baseline level of symptom burden and impact on quality of life, which is what we have done for POS. This also serves as a basis for assessing the impact of future therapeutic interventions for POS, using a standardized measure of symptom burden and quality of life.

When reading this paper, the question that kept coming up for me - Would we see these same differences in a comparison of persons with POS versus not in the general population, regardless of COVID infection? If so, then why is COVID at the center of this study? There are other possibilities for a control group, of which I would recommend two: persons with POS without a COVID diagnosis, or persons without POS and without a COVID diagnosis. Including both of these control groups would help us better understand if it is COVID that is in fact causing differences in the measures of interest, or if it is simply the POS. As the paper is currently designed, all we know is some of these measures differ for persons reporting POS compared to persons not reporting POS, which doesn't seem all that interesting or important.

We thank the reviewer for the opportunity to discuss these important aspects of our study design, to improve the quality and informative value of our study.

Regarding the first two questions, whether we would see the same differences in persons with POS vs those without in the general population and why COVID is at the center of our study – POS should be seen as a COVID-related phenomenon, like long COVID. POS is not an entity that existed previously in the general population. The main point of our study is to describe a group of individuals presenting with a unique spectrum of similar and inexplicable ocular symptoms that started emerging at ophthalmology clinics during and after the COVID pandemic, which we are defining for the first time as POS. The many confused doctors and dissatisfied patients led us to investigate this phenomenon further. Parallels can be made with long COVID, which was similarly an unknown entity prior to the pandemic and with features, biomarkers and therapies still being researched.

Regarding the suggested possibilities for control group, we again refer to our discussion above. POS does not exist separately from COVID infection; COVID-19 is a necessary precondition for developing POS. To give a related example, the well-established long COVID or PACS (post-acute Covid-19 syndrome) is a syndrome

comprised of a set of distinct clusters of symptoms that does not exist outside of the context of COVID, and where prior SARS-CoV-2 infection is a necessary precondition for development of PACS.

Regarding a control group of individuals without POS and without COVID diagnosis, it was not possible to assemble such a group of individuals during our study, for several reasons. First, both during and after the pandemic, we could not be certain if an individual did not have prior COVID-19, as many asymptomatic cases existed and increased over time and after vaccination. Testing control individuals objectively to rule out the presence of SARS-CoV-2 antibodies was also not feasible, as most of the Swedish population has been vaccinated once or several times, resulting in the presence of vaccine-associated antibodies that would confound antibody-based testing. For these reasons, the most applicable control group was a group of individuals with prior COVID-19 but without subsequent POS development. This has the advantage of isolating only the effect of POS development and not the prior presence of COVID-19 as a potential confounder.

However, it is possible to compare certain measures in our study with historic data taken prior to the COVID pandemic, to assess if COVID-19 or POS resulted in the observed changes. Pre-pandemic normative studies[1-3] have established the normal level of corneal subbasal nerve density as 18-23 mm/mm^2, which is in line with our control group, indicating the density reduction observed in POS differs from the general population. Similarly, the normal level of central corneal sensitivity by esthesiometry[4] is 58-60mm, again indicating a normal level in our control group and diminished level in POS. Mature central corneal dendritic cell density in healthy subjects has been reported[5,6] to be between 1.4 – 4.5 cells/mm^2, which is in line with our controls and this parameter was notably elevated in POS. These data indicate that our control group had values comparable to pre-pandemic values in the general healthy population, suggesting that POS but not prior COVID infection alone, resulted in the observed abnormal levels of inflammation and nerve presence and function. Normative pupil measurements, however, are sparse and difficult to obtain from the literature. Where values are published, they are difficult to compare, as they are dependent on the device used, operator, and ambient lighting conditions. While we controlled these factors in our study, we cannot directly compare these to literature values that used different techniques.

As readers may have the same question as the reviewer, we have now added our results of comparison with normative pre-pandemic data to the third-to-last paragraph in the main text and provided the relevant references listed below.

References

1. Parissi M, Karanis G, Randjelovic S, Germundsson J, Poletti E, Ruggeri A, Utheim TP, Lagali N. Standardized baseline human corneal subbasal nerve density for clinical investigations with laser-scanning in vivo confocal microscopy. Invest Ophthalmol Vis Sci. 2013 Oct 29;54(10):7091-102.

2. Dehghani C, Pritchard N, Edwards K, Vagenas D, Russell AW, Malik RA, Efron N. Morphometric stability of the corneal subbasal nerve plexus in healthy individuals: a 3-year longitudinal study using corneal confocal microscopy. Invest Ophthalmol Vis Sci. 2014 Apr 24;55(5):3195-9.

3. Tavakoli M, Ferdousi M, Petropoulos IN, Morris J, Pritchard N, Zhivov A, Ziegler D, Pacaud D, Romanchuk K, Perkins BA, Lovblom LE, Bril V, Singleton JR, Smith G,

Boulton AJ, Efron N, Malik RA. Normative values for corneal nerve morphology assessed using corneal confocal microscopy: a multinational normative data set. Diabetes Care. 2015 May;38(5):838-43.

4. Kotak, Sharon; Christina, Mini; Selvin, Satheesh Solomon T. Establishing the Normal Range of Corneal Sensitivity in an Indian Population Using the Cochet–Bonnet Esthesiometer. Current Medical Issues 18(3):p 170-174, Jul–Sep 2020.

5. Akhlaq A, Colón C, Cavalcanti BM, Aggarwal S, Qazi Y, Cruzat A, Jersey C, Critser DB, Watts A, Beyer J, Sindt CW, Hamrah P. Density and distribution of dendritiform cells in the peripheral cornea of healthy subjects using in vivo confocal microscopy. Ocul Surf. 2022 Oct;26:157-165.

6. Lagali, N.S., Badian, R.A., Liu, X. *et al.* Dendritic cell maturation in the corneal epithelium with onset of type 2 diabetes is associated with tumor necrosis factor receptor superfamily member 9. *Sci Rep* 8, 14248 (2018).

Finally, as the reviewer points out, as the study is currently designed, some measures differ for persons reporting POS compared to persons not reporting POS. This is precisely what we aimed to find, namely, objective measures related specifically to POS and not to COVID-19 generally. This is what both the ophthalmologists and patients have been seeking, i.e., objective criteria to confirm the presence of POS that establishes it as an entity with specific and measurable biomarkers that may provide insights as to its origin.

In addition to the above issue, there is way too much data included in the report and it is very easy to get lost. Good research has a more targeted research question, and it feels overwhelming to see all of this information together. Some items I was confused about:
1. Why are person measures between the two groups compared. They already self-selected into groups as having vision issues, so of course they differ in their self-reported vision.

The person measure scores are the standard method for reporting Rasch analysis results from the Catquest 9SF form. It was known that self-reported vision differed between groups at the outset; however, a quantitative and statistical measure of the level of deviation from controls is valuable and enables comparisons to be made. For example, we now know that based purely on visual function (ignoring factors in POS such as pain, photophobia, etc.), that POS visual disability is at the same level as patients just prior to cataract surgery.

2. Gender differences in symptoms are graphed, but I couldn't find mention of it in the text. Are these differences significant? If not, they should not be included. If they are, the text should mention this.

We thank the reviewer for pointing out this omission on our part. We have now revised the main text to indicate that after comparison of proportions of males and females with each symptom, no significant differences were found (Chi-square test with FDR threshold < 0.05). We also updated the Statistics part of the Methods accordingly.

3. Then there are a bunch of figures that I can't make much sense of. Are these all

important? Could they be in a table in order to digest easier?

We have taken care to include in the main manuscript only the most relevant figures that provide the data supporting our results, and refer the reader to the supplementary figures, not published within the main paper, for further information. We believe that this level of detailed and rigorous reporting is warranted in our study and conforms to the standard of the journal. Tabular data has been included in Supplementary Tables 1-5. Graphical data has been included in the main figures for ease of observing differences, distributions, and individual data points in a way that tabular numeric data cannot. We have, however, in response to a reviewer comment above, considerably simplified the graphical data for ease of digesting (i.e., simplified the boxplots).

4. Given the methodologic issues mentioned above, ROC curves are not appropriate.

Given the above discussion and responses, we believe the diagnostic information we provide in the study can be useful for other clinics in assessing the likelihood of POS in patients. At the ophthalmology clinic in our university hospital, the objective ocular measurements and ROC curves are used to assess the probability that a patient's symptoms are consistent with POS. This is of practical value in cases with a high suspicion of POS, as these patients can then be referred for further detailed tests (eg., strabismus) and to optometrists for specialized rehabilitation programs. We feel it is important that this potential diagnostic workflow is available for others, whether or not they choose to use it. Further, the ROC curves were used to define objective criteria for tear samples chosen for Western blot validation of our proteomics results, in response to the request from Reviewer #2.
